# Genome Analysis Coupled With Transcriptomics Reveals the Reduced Fitness of a Hot Spring Cyanobacterium *Mastigocladus laminosus* UU774 Under Exogenous Nitrogen Supplement

**DOI:** 10.3389/fmicb.2022.909289

**Published:** 2022-07-01

**Authors:** Mayuri Mukherjee, Aribam Geeta, Samrat Ghosh, Asharani Prusty, Subhajeet Dutta, Aditya Narayan Sarangi, Smrutisanjita Behera, Siba Prasad Adhikary, Sucheta Tripathy

**Affiliations:** ^1^Computational Genomics Lab, Structural Biology and Bioinformatics Division, CSIR Indian Institute of Chemical Biology, Kolkata, India; ^2^Academy of Scientific and Innovative Research (AcSIR), Ghaziabad, India; ^3^Department of Biotechnology, Siksha Bhavana, Visva-Bharati, Santiniketan, India

**Keywords:** hot spring cyanobacteria, nutrient and nitrogen stress, heat shock, nitrogen fixation, Hapalosiphonaceae

## Abstract

The present study focuses on the stress response of a filamentous, AT-rich, heterocystous cyanobacterium *Mastigocladus laminosus* UU774, isolated from a hot spring, Taptapani, located in the eastern part of India. The genome of UU774 contains an indispensable fragment, scaffold_38, of unknown origin that is implicated during severe nitrogen and nutrition stress. Prolonged exposure to nitrogen compounds during starvation has profound adverse effects on UU774, leading to loss of mobility, loss of ability to fight pathogens, reduced cell division, decreased nitrogen-fixing ability, reduced ability to form biofilms, reduced photosynthetic and light-sensing ability, and reduced production of secreted effectors and chromosomal toxin genes, among others. Among genes showing extreme downregulation when grown in a medium supplemented with nitrogen with the fold change > 5 are transcriptional regulator gene WalR, carbonic anhydrases, RNA Polymerase Sigma F factor, fimbrial protein, and twitching mobility protein. The reduced expression of key enzymes involved in the uptake of phosphate and enzymes protecting oxygen-sensitive nitrogenases is significant during the presence of nitrogen. UU774 is presumed to withstand heat by overexpressing peptidases that may be degrading abnormally folded proteins produced during heat. The absence of a key gene responsible for heterocyst pattern formation, patS, and an aberrant hetN without a functional motif probably lead to the formation of a chaotic heterocyst pattern in UU774. We suggest that UU774 has diverged from *Fischerella* sp. PCC 9339, another hot spring species isolated in the United States.

## Introduction

Cyanobacteria are highly diverse oxygenic phototrophs inhabiting terrestrial and aquatic ecosystems. They can also successfully thrive in extreme environments such as deserts, hot springs, and polar regions. These organisms are key primary producers maintaining our planet's biogeochemical cycles of nitrogen, carbon, and oxygen (Tomitani et al., 2006; Finsinger et al., 2008; Alcamán et al., 2017). Cyanobacteria form an integral part of thermal springs and are the center of attraction and attention for microbial ecologists due to their unique adaptation strategies (Tomitani et al., 2006). Characterization of thermophilic cyanobacteria has been extensively performed in alkaline siliceous hot springs of Yellow National Park (YNP), United States, particularly the thick mats of Octopus and Mushroom springs (Kees et al., 2022). Members belonging to the genera *Leptolyngbya, Fischerella, Mastigocladus, Synechococcus-like, Geomargarita, Gloeocapsa, Gloeocapsopsis, Oscillatoria*, and *Thermosynnechococcus* are predominant cyanobacteria occurring in hot springs spanning various ecoregions of the world (Amarouche-Yala et al., 2014; Alcamán et al., 2017; Jasser et al., 2022). A thermophilic cyanobacterium, *Mastigocladus laminosus* UU774, isolated from a hot spring, Taptapani, in India (19.3011° N, 84.2424° E) is discussed in the present study. The thermal taxon *Mastigocladus laminosus* widely occurs in hot springs of moderate temperature below 60°C and pH > 7.5 (Kaštovsk and Johansen, 2008). Most of the reported studies on this thermophile are based on its spatial distribution, genetic diversity, and structure of photosynthetic machinery. Phylogeographic examination suggested the existence of seven major lineages comprising a total of 23 haplotypes in *M. laminosus* (Miller et al., 2007), whereas the distribution of this thermophile in Asia revealed the presence of two lineages with different cell sizes (Soe et al., 2011). Moreover, x-ray resolved structures of phycobilisomes (Glauser et al., 1992) and c-phycocyanin (Schirmer et al., 1985) and cytochrome b6f (Kurisu et al., 2003) structures from *M. laminosus* were also discussed.

Highly developed diazotrophic cyanobacteria of the order Stigonematales (subsection V) are prevalent in hot spring microbial mat communities and perform primary production and reduction of molecular nitrogen to ammonia in these systems (Finsinger et al., 2008; Estrella Alcaman et al., 2015). Cyanobacteria harbors nitrogenases, an enzyme complex that is capable of fixing atmospheric nitrogen. Nitrogenases are highly sensitive to oxidative damage; therefore, many species of cyanobacteria have adapted to temporal nitrogen fixation, where photosynthesis occurs during the day and nitrogen fixation happens during the night. Many other species of cyanobacteria have evolved as a specialized structure, such as heterocysts, that provides spatial separation between oxygen and nitrogenases (Fay, 1992). In heterocysts, the oxygen concentration is lowered to protect the nitrogenase enzyme complex (Silverman et al., 2019). Heterocyst-forming cyanobacteria are monophyletic, and they are believed to have evolved only once about 2,000 million years ago (Tomitani et al., 2006). Heterocyst differentiation is very well-orchestrated with nitrogen step down, leading to the accumulation of 2-oxoglutarate, which regulates the global nitrogen regulator NtcA, triggering the nitrogen response regulator that ultimately triggers HetR, the transcription factor responsible for heterocyst differentiation (Kumar et al., 2010). HetR forms a dimer and has DNA-binding activity upon dimerization (Huang et al., 2004). The number and location of heterocyst formation are tightly regulated to conserve energy (Silverman et al., 2019). In geothermal systems, diazotrophic filamentous cyanobacteria are implicated in nitrogen fixation. *Fischerella thermalis* (also known as *Mastigocladus laminosus*) fixed maximum nitrogen and contributed significantly to the nitrogen and carbon inputs of thermal mats (Estrella Alcaman et al., 2015; Alcorta et al., 2019). A *Mastigocladus* strain showed an increase in the expression of the *nif* gene at 50°C, thus serving as a significant nitrogen fixer in Porcelana hot spring (Chile) (Alcamán et al., 2017). The ability to acquire sufficient nitrogen is an important fitness trait for all cells. In the nitrogen-limited, geothermally influenced stream of YNP, a population of *Mastigocladus laminosus* fixed atmospheric nitrogen with varying N2 fixation activities. Strains showing high nitrogen fixation performance are revealed to have a gene with a premature stop codon encoding disrupted regulatory histidine kinase, resulting in transcriptional rewiring (Hutchins and Miller, 2017). This thermophilic population is also known to show phenotypic differences in response to environmental nitrogen availability. One population exhibited heterocyst development under nitrogen-limited conditions, whereas other populations did not express heterocyst formation and nitrogen fixation in the presence of nitrogen (Miller et al., 2006). In another report studying the effects of a combined nitrogen source on *M. laminosus*, the organism underwent reduction in cell growth, photosynthesis, and pigment content upon exposure to nitrogen (Rajalakshmi N, 1985). A study reported the role of PatS, a heterocyst inhibitor, in heterocyst differentiation and spacing pattern of *M. laminosus*. The organism failed to form heterocysts under nitrogen deprivation upon the addition of extraneous PatS-5 (RGSGR, the pentapeptide motif that is responsible for inhibition) and could not survive (Antonaru and Nürnberg, 2017).

Here, an in-depth account of the adaptation of hot spring species under starvation in the presence and absence of an external nitrogen source is elucidated. We delineated a possible mechanism by which they cope with hot temperatures despite growing under laboratory conditions for more than a decade.

**Figure 1:**
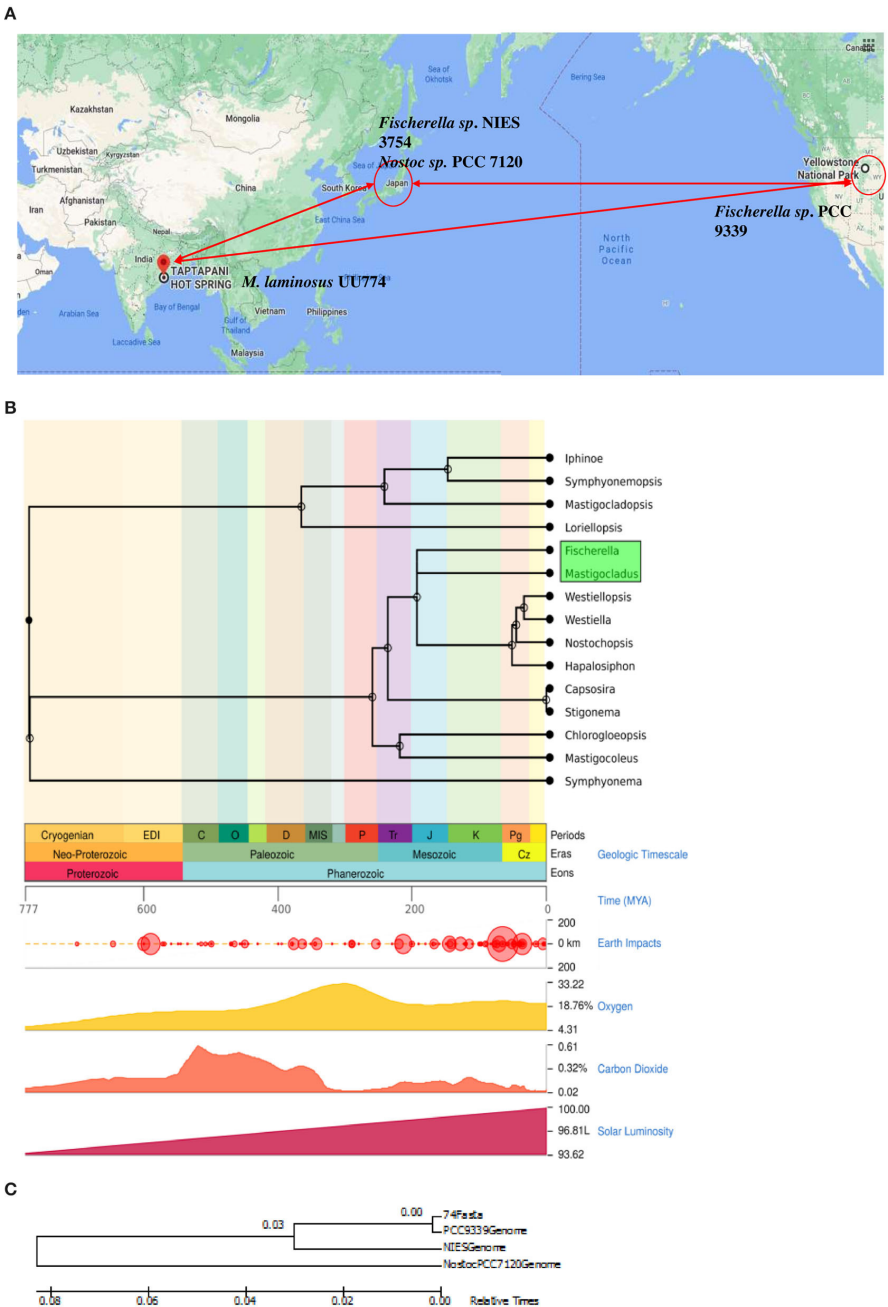
**(A)** Geographical location of the strains used in this study. The map was generated using google maps software (Google, n.d.). *Mastigocladus laminosus* UU774 was isolated in Eastern India and sequenced and analyzed by us, whereas the genomic data of PCC 9339, PCC 7120, and NIES-3754 were downloaded from Genbank. Hot spring species *Fischerella* sp. NIES-3754 was isolated in Japan and *Fischerella* sp. PCC 9339 was isolated in Yellowstone National Park, CA, United States. The non-hot spring species *Nostoc* sp. PCC 7120 used for comparison was isolated in Japan. **(B)** Divergence time using an evolutionary time scale between *Mastigocladus laminosus* UU774 and the *Fischerella* species PCC 9339 was calculated using the Timetree.org database and was estimated to be ~198 million years (MY). The calculation is conducted on the basis of a method described in Kumar et al. (2017). **(C)** The phylogenetic tree was generated from UU774, PCC 9339, and NIES-3754, and PCC 7120 was computed using Microbializer (Avram et al., 2019).

Using second- and third-generation sequencers, we report the genome of UU774 with a Genbank accession number of MNPM00000000. An extensive comparative study on this assembled genome with two other genomes of hot spring origin from diverse geographical locations, e.g.; *Fischerella* sp. PCC 9339 from Yellow Stone National Park, United States (Shih et al., 2013) and *Fischerella* sp. NIES-3754 from Suwa-shrine, Japan (Hirose et al., 2016), was performed to bring insights into the origin of *M.laminosus* UU774. PCC9339 is the closest known strain of UU774 and is a permanent draft, whereas NIES-3754 is a complete genome. The complete genome of *Nostoc* sp. PCC 7120 is also included for comparison purposes (Kaneko et al., 2001). These species will henceforth be referred to as UU774, PCC 9339, NIES-3754, and PCC 7120. Based on time estimates as described in a previous study (Hedges et al., 2015), UU774 and PCC 9339 diverged from each other 200 million years ago, and this lineage diverged from Nostocales some 250 million years ago (Kumar et al., 2017) (Figure 1).

## Materials and Methods

### Culture Collection and Microscopy

UU774 was isolated from a hot spring in the Indian state of Odisha (19.3011°N, 84.2424°E). The pH of the hot spring is slightly alkaline in the range of 8 and the on-site temperature range is ~50°C. Liquid cultures of UU774 were grown in a BG11 (Rippka et al., 1979) medium without sodium nitrate (BG11N-) (Supplementary Table 1) at 25°C, with a light intensity of 3,000 lux maintaining a 16-h light/8-h dark period cycle and with daily manual shaking. Manual shaking involves swirling the culture flasks in a circular motion for few seconds two times in a day. Light microscopic images were generated using an EVOS microscope (EVOS XL Imaging System, Cat no; AME3300 Life Technologies Corporation, Bothell).

### Whole Genome Sequencing Using Illumina and Oxford Nanopore

DNA isolation and purification were carried out using Thermo Scientific GeneJET Plant Genomic DNA Purification Mini Kit (Cat. No: K0791 for 50 preps). The Illumina Hiseq paired-end library had a 300-bp insert size and the mate-pair library had a 3-kb insert size. DNA was quantified using Qubit® 3.0 Flourimeter (Thermo Fisher Scientific, Life Technologies Holdings Pte. Ltd., Malaysia) for in-house nanopore sequencing using ligation-based LSK-108 chemistry.

### Processing Sequencing Reads and Hybrid Genome Assembly

Reads from the paired-end library were analyzed using FastQC (Bioinformatics, 2011), followed by adapter trimming and discarding poor quality and shorter reads having a length of <100 bp. Reads from the mate-pair library were processed using NxTrim (O'Connell et al., 2015). Nanopore reads were incorporated into the assembly after conducting an error correction based on FMLRC (Wang et al., 2018).

The SPAdes genome assembler (v3.11.1) with nine different k-mers (25 to 97 with step size 8) was used for genome assembly (Bankevich et al., 2012). Scaffolds with a length shorter than 1,000 or a k-mer coverage <10 were removed from the final list and were super scaffolded using BOSS (Luo et al., 2017).

### Genome Analysis and Protein Structure Prediction

The genome was further analyzed using CheckM (Parks et al., 2015) to assess its completeness and degree of contamination. BUSCO (v4.0.6) analysis was conducted to determine the presence of complete core genes using database version 10 (Simao et al., 2015). Species boundary demarcation was carried out using pyani (Pritchard et al., 2016) to calculate average nucleotide identity (ANI) and TETRA nucleotide values, and CompareM was used to calculate average amino acid identity. Data visualization and plot generation were carried out using RStudio (R. T. and Core, 2013). For plasmid prediction, plasFlow (Krawczyk et al., 2018) was used. PHASTER (Arndt et al., 2016), MetaPhinder (Jurtz et al., 2016), and Seeker (Auslander et al., 2020) were used to determine the presence of phage sequences. IS elements, CRISPR and Cas, were detected using CRISPRFinder and ISsaga (Varani et al., 2011), respectively (Grissa et al., 2007). PGAP (Tatusova et al., 2016) and PROKKA (Seemann, 2014) were used for genome annotation. A circular map of UU774 was generated using DNAPlotter (Carver et al., 2009). BlastKoala (Kanehisa et al., 2016) and Kofam KOALA (Aramaki et al., 2020) were used for assigning KO IDs to protein sequences.

Pathway analysis and ortholog prediction were carried out with KEGG KAAS (Moriya et al., 2007) and Orthovenn2 (Wang et al., 2015), respectively. A Jupiter plot that uses Ragtag (successor of Ragoo) (Alonge et al., 2019) was created for comparing the genomic segments of UU774 and PCC 9339. An evolutionary analysis of Stigonemataceae was performed using the Timetree database (Kumar et al., 2017). MCscanX (Wang et al., 2012) was used for the prediction of collinearity, dN/dS analysis, and tandem duplication of genes.

The structure of the HetN protein of 4 organisms was predicted using AlphaFold v2.1.0 (Jumper et al., 2021). The confidence of the predicted models was evaluated using a per-residue confidence score by the predicted local-distance difference test (pLDDT) and the predicted aligned error (PAE) (Supplementary Figure 1). The predicted 3D structures were further superimposed and visualized using PyMOL 2.5.2.

### Differential Transcript Expression Analysis

**Figure 2:**
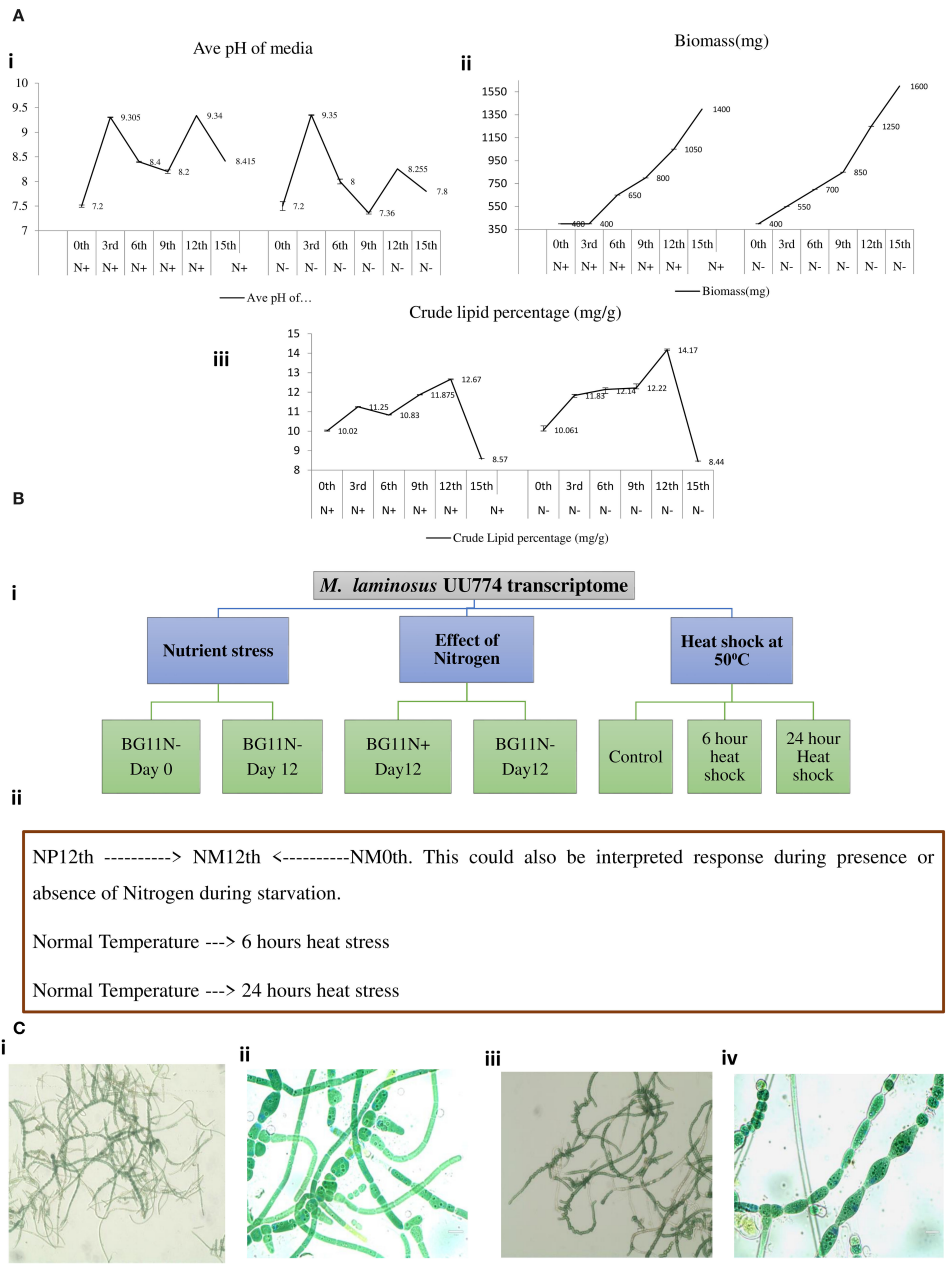
A pilot experiment to determine the time point for metabolic switch. **(A)** (i) Cultures were grown in BGN– and BGN+ media, and pH change of the media was noted. On the 3rd day of growing the cultures, there was a sharp peak for cultures grown in both the N– and N+ media. This may be correlated with increased metabolism. On day 9, there was a fall in the pH, but it rose again on the 12^th^ day before declining further. All the experiments were repeated 3 times. **(A)** (ii) Continuous increase in biomass was noted when the organism was grown in the N+ and N– media. The increased growth was steady until day 12 of the culture. The growth curve continued to increase until the 15th day presumably because of the presence of living and dead biomass. The biomass for cultures grown in the N– condition was consistently more than that for cultures grown in the N+ condition. **(A)** (iii) Crude lipid content in the culture was estimated continuously from days 0 to 15. There was an increase in the crude lipid content on the 3rd day followed by a drop on the 6th day and a subsequent rise on the 12th day, subsequently declining quantities of the lipid content till the 15th day and beyond. We suggest that the increase in lipid content is due to the rapid growth of the organism in the culture. After complete depletion of nutrients on the 12th day, lipids are utilized; hence, there seemed to be a decline in quantity. The crude lipid content also showed increased levels under N– conditions than under N+ conditions. **(B)** Experimental design for RNAseq experiments. (i) Nutrient stress was calculated between cultures grown on the 0th day (6 h) and the 12th day in N– media. The experiments were replicated two times and each time with 2 flasks. For heat stress, the cultures were inoculated into N– media and were kept at 50^0^C for 6 h vs. cultures kept at 50^0^C for 24 h. For nitrogen stress, cultures were grown for 12 days under N– conditions and were compared with cultures grown under N+ conditions. (ii) A schematic representation of the experimental conditions used for RNAseq: NM0th refers to a culture grown in media without a nitrogen source (nitrogen minus) for the 0th day; NM12th refers to a culture grown in media without a nitrogen source (nitrogen minus) for the 12th day; NP12th refers to a culture grown in media with a nitrogen source (nitrogen plus) for the 12th day; heat stress for 6 and 24 h are the cultures kept at 50^0^C. **(C)** Cultures grown in N– media (i and ii) and N+ media (iii and iv) were visualized under an EVOS Life Technologies microscope at 20× (i and iii) and 100× (ii and iv) resolutions.

Our pilot experiments involving changes in pH, biomass, and crude lipid content with respect to the number of days in culture suggested that some drastic physiological changes happened on the 12th day of the culture. Therefore, we took the 12th day of the culture as the time to study the effect of starvation (Figure 2A). Nutrition stress (starvation) was studied by comparing cultures grown for the 12th day in the N-media condition with cultures grown on the 0th day. Furthermore, the effect of starvation on the presence and absence of nitrogen by comparing the expression pattern between 12th day N+ and N-grown cultures was studied. The effect of heat stress (cultures grown at 50°C temperature) after 6- and 24-h intervals on N media with cultures grown at 25°C was studied. All the experiments were performed in duplicates. The schematic diagram representing the experimental conditions is provided in Figure 2B.

RNAseq data were generated using the Illumina Nextseq platform. Adapter sequences were removed from raw RNASeq reads using Fastp (Chen et al., 2018), and reads with lengths below 75 bp were removed. The RNAseq reads were aligned back to the UU774-assembled genome using Bowtie2 (Langmead and Salzberg, 2012). Quantification of the transcript expression was carried out using Kallisto (Bray et al., 2016). Differential expression analysis was performed using DEseq2 (Love et al., 2014). Transcripts with log2Fold change (−2 to +2) and *p*-value ≤ 0.05 were considered for further analysis. We created a workflow for prokaryotic transcript expression profiling using Python and R language and implemented it with the python-based Luigi workflow manager.

### Validation of Selected Transcripts by RT-PCR

RNA extraction from UU774 using 200 mg of the starting material was performed following a modified CTAB (Cetrimonium bromide, Hexadecyltrimethylammonium Bromide, Cat no: H5882-100G, Sigma-Aldrich, Product of China) method where a buffer comprising CTAB, β-mercaptoethanol, and phenol: chloroform (1:1) at a 1:0.2:1 ratio. Primers were designed using NCBI primer- BLAST (Ye et al., 2012) (Supplementary Table 1:Primers). The extracted RNA transcripts were converted into cDNA using a Verso cDNA synthesis kit (Cat AB1453A; Invitrogen), and RT-PCR for the selected transcripts was performed using a FastStart Essential DNA Green Master Mix, Roche diagnostics, Mannheim, Germany (Cat. No. 06402712001; Roche) in LightCycler96 (Roche Diagnostics India Pvt. Ltd., Kolkata, India). For each of the three biological samples, three technical replicates were used. The quantitative expression values of the transcripts were converted into a log2 scale for comparing the fold change value with that of the RNAseq analysis.

## Results and Discussion

### Highly Compact UU774 Genome With 99.52% Completeness Reveals That 7.2% of the Genes Are Overlapping With Each Other

The Illumina sequencing generated 9,464,837 paired-end reads with 101-bp length at 137X coverage and 3,019,370 mate-pair reads with 101-bp length at 42.9X coverage. Finally, 9,464,837 cleaned paired-end and 1,350,962 mate-pair reads were retained. Nanopore reads (N50 length of 19,806,139 and 7332 reads) were error-corrected using Illumina sequences and the FMLRC tool prior to incorporating them into the assembly pipeline. The final assembly with 99.52% completeness and 0.24% contamination resulted in 52 scaffolds, 210,018 bp N50, and 9 L50, as analyzed with Quast (Gurevich et al., 2013). The genome had 80% of its entire length predicted to be coding with 5,827 CDSs. Out of the 5,827 CDSs, 419 overlapped with each other, with the highest overlap of 144 bases and the lowest of 1 base. About one-third (138 genes; 33%) of the genes that overlapped had 4 bases. The percentage of genes with 4-base overlap in the species PCC 9339, NIES-3754, and PCC 7120 was 35, 39, and 50%, respectively, making them the majority among the other genes that overlapped. In bacteria, overlapping genes provide transcriptional and translational regulation and often are indicators of rapid evolution and adaptation (Luo et al., 2013). The overlap by 4 bases provides an alternate frame for translation. The UU774 genome does not have compartmentalization into gene sparse and gene dense region and hence is not a two-speed genome (Table 1; Supplementary Table 1; Supplementary Figures 2, 3).

**Table 1:** Genome statistics of UU774, PCC 9339, NIES-3754, and PCC 7120.

**Genomes**	***Mastigocladus laminosus* UU774**	***Fischerella* sp. PCC 9339**	***Fischerella* sp. NIES-3754**	***Nostoc* sp. PCC 7120**
Genbank accession no.	MNPM02000001	ALVS01000000	AP017305	BA000019
Genome size and gap length	6,911,113 bp; 24,331 bp	8,008,287 bp; 8,238 bp	5,826,863 bp; 0 bp	7,211,789 bp; 0 bp
GC content	40.21%	40.16%	40.98%	41.27%
No. of CDS	5,827	6,650	4,935	6,012
No. of tRNAs	44	49	44	70
CRISPR arrays	16	14	4	11
No. of pseudogenes	398	331	238	204
Total coding length and percentage of coding region	5,530,423 bp (80%)	6,114,132 bp (76%)	4,637,184 bp (79.5%)	5,939,363 bp (82%)
Average gene length	949.1 bp	967.57 bp	939.65 bp	969.37 bp
Average intergenic distance	234 bp	240 bp	236.47 bp	210.66 bp
Number and percentage of overlapping genes	419 (7.2%)	376 (5.6%)	282 (5.7%)	363 (6.03%)
BUSCO completeness (complete Buscos; complete Single Buscos; Complete Duplicate Buscos; Fragmented Buscos; Missing Buscos	99.1% (766; 757; 9; 2; 5)	99.2% (767; 754; 13; 1; 5)	99.8% (772; 767; 5; 1; 0)	98.4% (761; 761; 0; 12; 0)
No. of plasmids	5	6	2	6

### Unusual Nucleotide Composition of a Hitherto Unknown and Indispensable Scaffold_38

A comparison of the nucleotide composition of scaffolds of UU774 among themselves indicated a composition for scaffold_20, scaffold_35, scaffold_38, scaffold_42, scaffold_46, scaffold_48, and scaffold_52 that was different from that of the rest. Scaffold_20 was flagged as a contaminant after comparing this sequence with the Genbank database and checking the expression patterns of the genes encoded in this scaffold. Furthermore, scaffold_20 had very high sequence similarity to *Sphingomonas* bacteria that was earlier undetected with Genbank's contamination filter. PlasFlow predicted scaffold_35, scaffold_42, scaffold_46, scaffold_48, and scaffold_52 as plasmids (Supplementary Figure 4; Supplementary Table 3:UU774 plasmids).

Scaffold_38, a 44,716-bp-long genomic segment containing 52 genes (Supplementary Table 4: Genbankgff), has a very unusual composition compared to the rest of the genomes of UU774 and with other genomes on Genbank with <5% identity. While MetaPhinder and PHASTER (Arndt et al., 2016) failed to predict the origin of this sequence from a Phage, a SEEKER score of 0.64 (value > 0.5 could be of phage origin) indicated that this scaffold may have some phage components. Conversely, PHASTER predicted two other regions of a genome of phase origin (e.g., scaffold_0:68940-77915; scaffold_6: 61430-67557) (Supplementary Table 3: Phage elements).

Except for a gene from scaffold_38, BLD44_028385, which forms a cluster with BLD44_023160 from scaffold_21 (ClusterID: 5587), meaning that they have probably originated from the same source, all other genes are singletons (Supplementary Table 5). Another gene, BLD44_028430, (UniProtKB: P19821 IPR002298) is predicted as coding for DNA polymerase A; it is thermostable and is constitutively transcribed (Supplementary Table 4: Normalized count), indicating its indispensable nature. Genes from BLD44_028500 to BLD44_028560 have been annotated with an unknown function and are highly downregulated in N+ conditions as confirmed by qRT-PCR (Supplementary Table 1: RT-PCR validation and Supplementary Figure 5). The gene BLD44_028540 in that cluster is annotated as STIVB116 (Sulfolobus Turreted Icosahedral Virus infects Sulfolobus species found in the hot springs of Yellowstone National Park) is missing in the hot spring species PCC 9339. Certain genes in that cluster that have some predicted functions including murein hydrolase activator NlpD, carbohydrate-binding proteins, genes for plasmid stabilization and segregation, genes for making actin filaments (parM/stbA BLD44_028600; IPR022389), and Phage Major capsid protein (BLD44_028570) are highly downregulated during the N+ condition. In the entire scaffold_38, 44 genes (~84%) show significant downregulation during prolonged nitrogen exposure under starvation.

From similarity studies, it appears that scaffold_38 contains genes of chromosomal, plasmid, and phage origins in an assorted manner. It is unclear whether these genes are acquired after UU774 diverged from PCC 9339 some 198 million years ago (Figure 1). While non-diazotrophic cyanobacteria undergo severe stress during nitrogen starvation that leads to chlorosis and proteolytic degradation that leads to dormancy (Neumann et al., 2021), with complex multi-cellular forms such as UU774 develop heterocysts. The cassettes of genes in scaffold_38 with the upregulation in the nitrogen depleted condition during starvation are possibly an indication that it is imparting adaptive advantages and is possibly indispensable.

### Multiple Genes in UU774 Show Ancient Acquisition Prior to Species Migration

The gene annotated as putative “functional group II intron reverse transcriptase/maturase” (BLD44_007940) is downregulated in the N+ condition and is absent in all known cyanobacteria species except for PCC 9339, where it is present in the plasmid JH992892.1 as a pseudogene (Supplementary Table 6). Interestingly, a BLAST search with Genbank only points to the presence of a rudimentary copy of this gene in *Fischerella* sp. NIES-4106 plasmid2 DNA. Possibly, this gene is integrated into the genome of UU774 before species diversification. Subsequently, it was possibly ameliorated while the copy in PCC 9339 lost its function.

A single copy pseudo-gene, Glutamate racemase (implicated in peptidoglycan biosynthesis) (May et al., 2007) with similarities to transglutaminase is present in a cluster containing hetL/patU in scaffold-14 of UU774 and is pseudogenized in PCC 9339 as well as in UU774 by a stop codon exactly in the 13th position (Supplementary Figure 6). This gene is functional in both PCC 7120 and NIES-3754. Transglutaminase is regulated by illumination time. It is crucial for chloroplast bio-energetics and has a photoprotective role in PSII systems (May et al., 2007). Altered genes related to light harvest and bio-energetics in both PCC 9339 and UU774 could indicate their possible adaptation toward changed light conditions before species migration.

The putative ATP-dependent Clp protease is functional in UU774 (BLD44_024170). However, it is absent in both NIES-3754 and PCC 7120 and is pseudogenized in PCC 9339 (RS38885). Clp protease proteolytic subunits, which are involved in misfolded protein degradation, might be relevant in heat tolerance. This class of Clp protease only has some similarities to *Fischerella* sp. NIES-4106. Our comparative genomics analysis using 45 Hapalosiphonaceae members suggests that NIES-4106 is relatively closer to UU774 and PCC 9339 and that they might have shared a common ancestry (Supplementary Figure 7A).

Among the genes that have undergone pseudogenization (Supplementary Table 4: Genebankgff), the major class is transposase (98/331 in PCC 9339 and 79/398 in UU774). PCC 9339 has a large genome (~8 MB). The most likely cause for this may have been transposase-mediated genomic expansion. Among all Hapalosiphonaceae members, PCC 9339 and NIES-4106 have the highest number of IS elements (Supplementary Figure 7B). We hypothesize that PCC 9339 and UU774 have acquired several genes by transposon-mediated insertions. Subsequently, due to speciation and adaptive purifying selection, the transposons had probably lost function and were pseudogenized.

### Gene Acquisition, Duplication, and Genomic Shuffling for Adaptive Advantage in UU774

Of the total tandem duplicated genes, PCC 7120 and PCC 9339 have the highest numbers, e.g., 88 each. NIES-3754 and UU774, on the other hand, have 56 and 39 tandems duplicated genes, respectively (Supplementary Table 5: Tandem duplication). In UU774, 3 out of 39 pairs of duplicated genes are chemotaxis related. We have also found that the largest number of chemotaxis-related genes is found in UU774 (Rajalakshmi N, 1985) compared to PCC 9339 (Silverman et al., 2019), NIES-3754 (Soe et al., 2011), and PCC 7120 (Kees et al., 2022), indicating that UU774 is more mobile.

Among the most notable pair of duplicated genes in UU774 that have undergone upregulation under starvation conditions is aminopyrrolnitrin oxygenase PrnD. This gene is present in duplicate copies in plasmid JH992893 of PCC 9339 (WP_01730 8631.1 and WP_017308637.1), but it is present in 4 copies in UU774 (BLD44_006720, BLD44_006725, BLD44_006730, and BLD44_006735). The gene prnD encodes antifungal antibiotic pyrrolnitrin and is absent in NIES-3754 and PCC 7120. Genes containing a cupin-like domain (BLD44_013180 and BLD44_013185), alcohol acetyltransferase genes (BLD44_018485, BLD44_018490), phthiocerol/phenolphthiocerol synthesis polyketide synthase PpsEs (BLD44_030080 and BLD44_030085), and spore wall maturation proteins (BLD44_027385 and BLD44_027390) are tandem duplicated in UU774, implicating adaptive acquisition (Supplementary Table 5: Ortholog of all clusters; tandem duplication file).

A novel apoptotic protease-activating factor (BLD44_008170, BLD44_008165) and a high light-inducible protein (BLD44_028735, BLD44_028740) are duplicated and upregulated during the N+ condition. A remote homolog of BLD44_008170 is present in PCC 7120 but is absent in PCC 9339 and NIES-3754.

The type IV pilus-twitching motility protein-encoding gene pilT (BLD44_023445, BLD44_023450), biofilm formation gene (BLD44_024400, BLD44_024405), and phototactic response regulators encoding patA (BLD44_011150, BLD44_011155) are downregulated in UU774 during the N+ condition, indicating their reduced ability to sense light in the N+ condition.

Tandem duplicated genes occur in bacteria as a result of retro-transposition activities and may add a dosage effect on the organism if retained for long (Weissenbach et al., 2017). The genes duplicated in UU774 are functional and involved in chemotaxis and light harvest, and they avert fungal pathogens, providing multiple adaptive advantages to the organism. Moreover, the most predominant cluster of proteins (out of 2,925 clusters in the 4 species studied) containing 34 members codes for a linear gramicidin synthase subunit D (uniprot id: Q70LM4) that may be important in defense response. Gene re-arrangements due to IS5 family transposase activities leading to synteny breakpoints are evident in several places (scaffold_4: BLD44_006515, BLD44_006545, BLD44_006770, BLD44_006810, BLD44_006860, and BLD44_006970) (Supplementary Figures 8, 9).

### Altered Functional Motifs in Cognate Heterocyst Inhibitor HetN in UU774 May Be the Reason for Chaotic Heterocyst Pattern Formation

**Figure 3:**
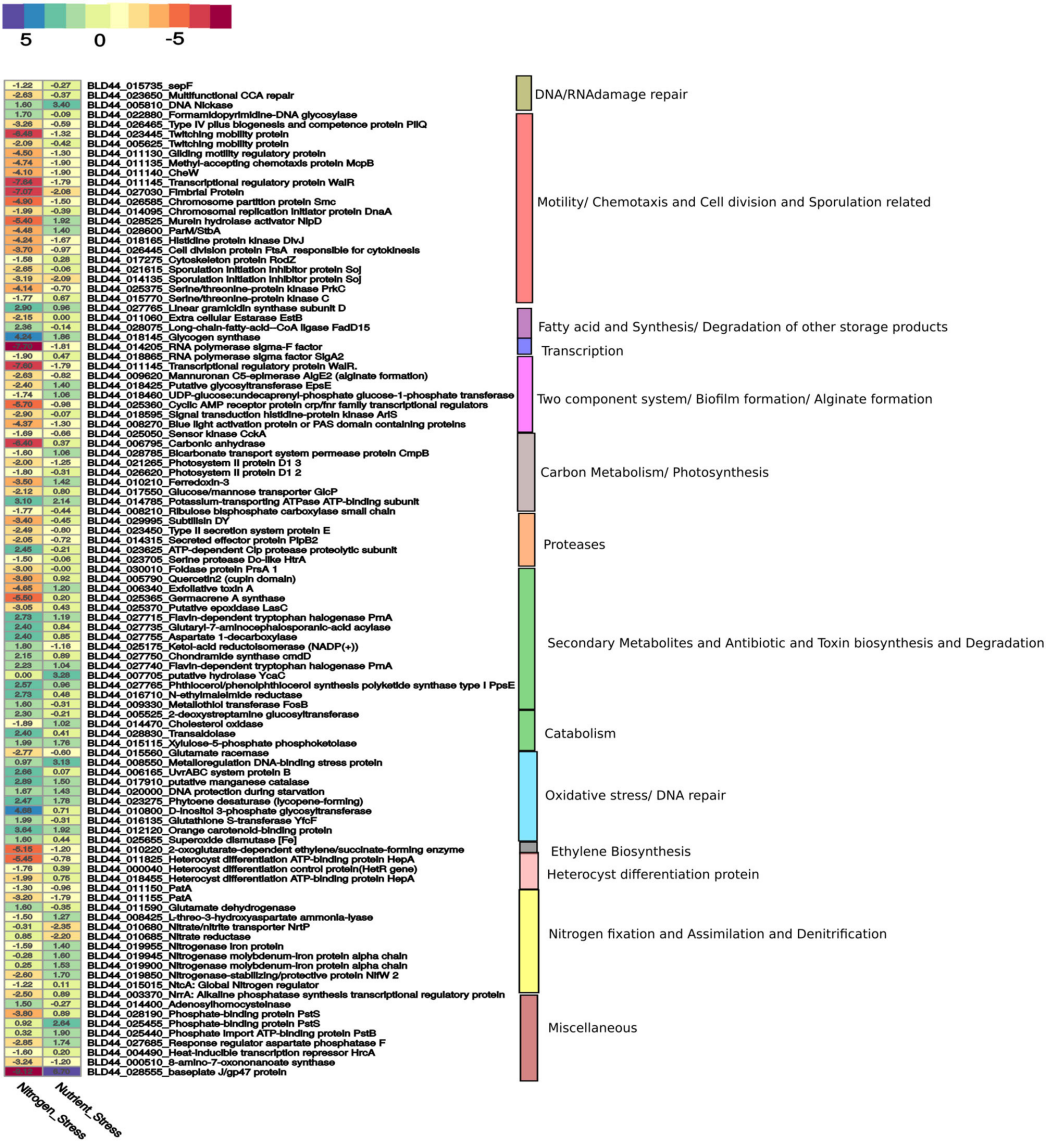
A curated list of genes taken from Supplementary Materials is represented here. The genes for nitrogen stress are represented with N– condition as the reference. For nutrition stress, the 0thday is used as a reference and compared with the 12th day culture under the N– condition. The upregulation and downregulation of the genes are shown in a color-coded heatmap. The gene categories are represented on the right panel.

**Figure 4:**
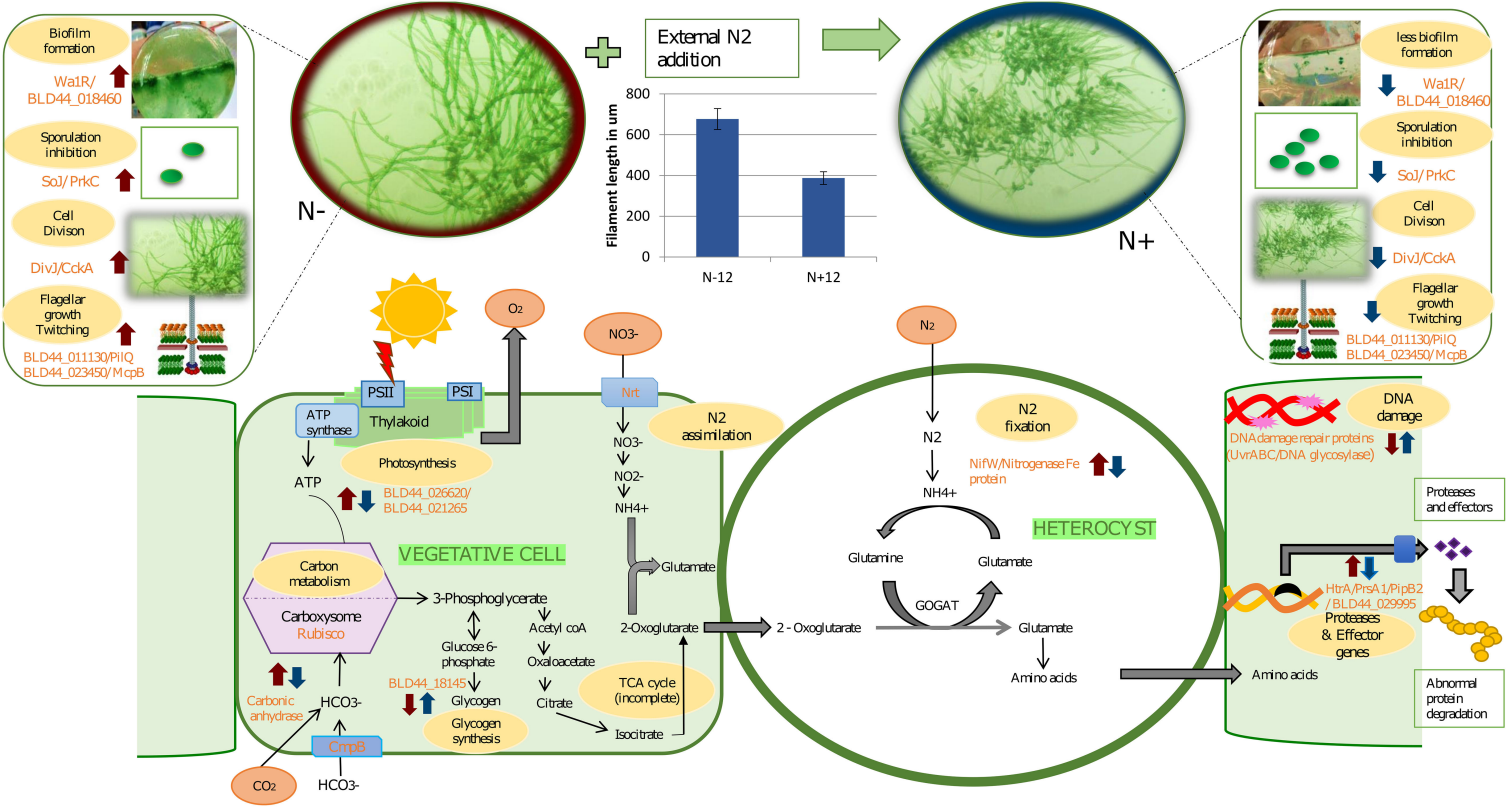
A graphical abstract containing some of the events taking place during prolonged exposure to nitrogen under depleted nutrition. The blue arrows indicate response due to the N+ condition compared to the N– condition on the 12th day of culture. Red arrows indicate mean response due to starvation on the 12th of N– grown cultures with the 0th day in N– condition. In other words, a blue arrow indicates the effect of long-term nitrogen exposure and a red arrow indicates the effect of starvation under N–. In the vegetative cells, during the N+ condition, there is a reduced carbon concentrating mechanism due to the downregulation of carbonic anhydrase. Complementing this, there is a downregulation of photosystem genes and hence reduced photosynthetic activities and slow growth. Slow growth triggers the upregulation of storage products such as glycogen that is upregulated during N+ condition. Among the other genes upregulated because of N– are biofilm (as seen in the image where a layer is formed at the bottom of the flask in N– condition), higher mobility in N-, and longer filaments, among others. Other genes that are affected are secretory proteins that protect the organism from predators and genes involved in DNA damage protection. We also show the increased filament length during the N– condition compared to the N+ condition.

Heterocyst formation is a tightly regulated process and the patterns are regular, as seen in PCC7120 (Kumar et al., 2010). Contrary to PCC7120, heterocyst formation was irregular in UU774. We manually curated 20 genes in UU774 responsible for the signaling and biogenesis of heterocysts. The global nitrogen regulator, ntcA (BLD44_015015), the nitrogen response regulator, nrrA (BLD44_003370), the key transcriptional regulator hetR (BLD44_000040), patB (BLD44_010215), members of the hepA family (BLD44_018455, BLD44_011825, BLD44_018455, and BLD44_018460) are upregulated in the N– condition and remain unperturbed during nutrient stress, while the remaining heterocyst genes have insignificant expression changes (Table 2; Figure 3). Although heterocyst differentiation is an early event, understandably, upregulation of nrrA and hetR during N– condition means an increased signal for heterocyst formation. We show microscopically that the number of heterocysts present in N– cells is higher than in N+ cells. The length of the vegetative filaments is larger in N– filaments than in N+ filaments (Figures 2C, 4; Supplementary Figure 10; Supplementary Table 1: Heterocyst count and filament length).

**Table 2:** Curated genes involved in heterocyst biogenesis and function in UU774, PCC 9339, NIES-3754, and PCC 7120.

***Nostoc* sp. PCC 7120**	***Mastigocladus laminosus*UU774**	***Fischerella* sp.PCC 9339**	***Fischerella* sp.NIES-3754**
hetC (BAB74516)	BLD44_008735 Annotated as Alpha-hemolysin translocation ATP-binding protein HlyB	WP_017313034	BAU07621
hetL (BAB75439) Annotated as pentapeptide containing protein	BLD44_012360 (remote to all) Annotated as penta peptide containing protein	WP_081594415	BAU04694
hetF (BAB73429)	BLD44_018745 Annotated as CHAT domain containing protein	WP_017309824	BAU05311
hetN (BAB77057.1) (annotated as Keto acyl reductase)	BLD44_016650 Annotated as SDR family NAD(P)-dependent oxidoreductase	WP_017313163	BAU07447
hetP (BAB74517) Homologs:BAB74601, BAB73629, BAB74933)	BLD44_008725 (heterocyst differentiation protein)	WP_017313032	BAU07618
hetR (BAB74038)	BLD44_000040	WP_017311371	BAU06403
hetZ (BAB77623)	BLD44_015585	WP_017312463	BAU05521
patU (BAB77625) Frameshifts	BLD44_015590 patU	WP_017312464	BAU05522 Intact transglutaminase
hepA (BAB74534)	BLD44_018455 Annotated as ABC Transporter	WP_017312250	BAU08665
hepC (BAB74533) Glycosyltransferase	BLD44_018460 Annotated as Sugar Transferase	WP_017312251 Annotated as Sugar Transferase	BAU08664
hepB (BAB75397) Heterocysts envelope polysaccharide synthesis protein	BLD44_009575 Annotated as Glycosyl Transferase Protein	WP_026081937 Annotated as Glycosyl Transferase Protein	BAU05904 Heterocyst envelope polysaccharide synthesis protein
hepK (”BAB76195) Annotated as two-component sensory histidine kinase	BLD44_021285 Annotated as HAMP domain-containing protein	WP_017312085	BAU08595 Annotated as Integral member Histidine kinase
patS (BAB74000)	Not found	Not Found	Not Found
patA (BAB72479)	BLD44_017940	WP_026081894	BAU06726
patB (BAB74211) Transcriptional regulator	BLD44_010215 helix-turn-helix domain-containing protein	WP_017313227 helix-turn-helix domain-containing protein	BAU06834 Transcriptional regulator PatB
patD (BAB77916)	BLD44_011395	WP_017312382	BAU06941
patN (BAB76511)	BLD44_011885 Heterocyst differentiation-related protein	WP_017308834 Hypothetical protein	BAU06901 Heterocyst differentiation-related protein
hep_A1 (BAB72541)	BLD44_011825	WP_017308846	BAU04614
hep_A2 (BAB74534)	BLD44_018455	WP_017312250	BAU08665
ntcA (BAB76091) Global nitrogen regulator	BLD44_015015	WP_017312798	BAU08821
nrrA (BAB76011) Nitrogen response regulator	BLD44_003370	WP_026081993	BAU04337

The heterocyst differentiation protein hetP converts the vegetative cells into heterocyst by recruiting hepA genes that produce polysaccharides essential for making thicker cell walls. The hetP ortholog (BLD44_008725) in UU774 is not significantly perturbed during nitrogen stress or in nutrient stress. Heterocyst commitment occurs during the 9–14 h of nitrogen depletion (Videau et al., 2016). Since gene expression was quantified on the 12th day of the culture, the expression changes might not have been captured. We also postulate that, even in the absence of a detectable pattern formation mechanism, heterocyst differentiation occurs in a controlled manner, leading to more numbers of heterocysts in N– media than in N+ media. In PCC 7120, there is one hetP, and 3 of its homologs share the same functional domain and act upon each other in an epistatic manner (Videau et al., 2016). On the contrary, in UU774, PCC 9339, and NIES-3754, only one copy of the hetP gene is present. The dN/dS ratio suggests that the hetP gene is under strong purifying selection (Supplementary Figure 11A). However, the hepA family of genes responsible for the production of the polysaccharide capsule of heterocysts are amply upregulated during N– condition even if it is at a later time point, e.g., on the 12th day.

**Figure 5:**
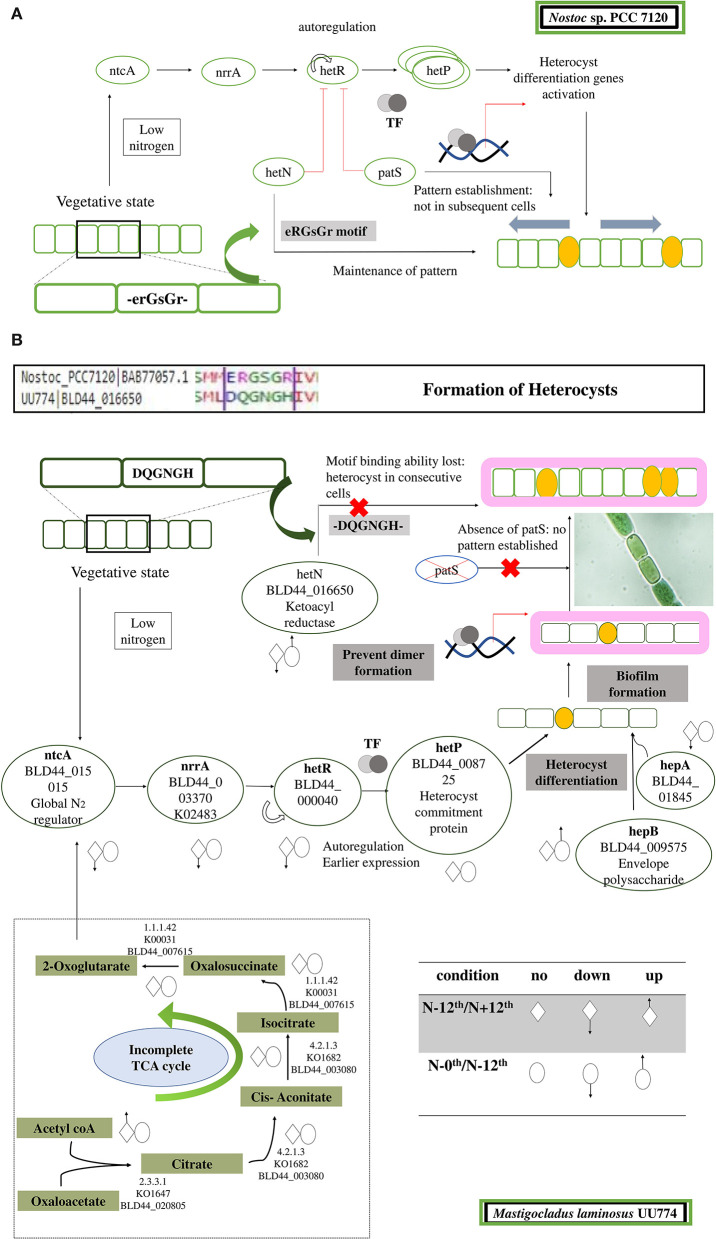
Cartoon depicting steps leading to the formation and regulation of heterocyst formation. **(A)** In PCC 7120, during nitrogen step down, a rapid rate of respiration is observed, leading to accumulation of 2-OG. Sensing 2-OG accumulation, a global nitrogen regulator ntcA signals a nitrogen response regulator NrrA that subsequently signals HetR. HetR is a master transcription regulator and is auto-regulated. In addition, there are few other disrupters of HetR that prevent the dimerization of HetR and subsequently turn it off. The disrupters are PatS and HetN. Both of these proteins have a DNA-binding motif, ERGSGR, that binds to HetR and regulates it. This helps in the patterned formation of heterocysts. After HetP is triggered, heterocyst formation takes place. **(B)** In UU774, PatS is missing and the ERGSGR motif of HetN is replaced by the DQGNGH motif and most likely leads to a lack of binding affinity to HetR. In UU774, patterned heterocyst formation is not observed, as is the case with PCC 7120. We hypothesize that, due to the lack of binding motif and absence of PatS, sometimes contiguous heterocyst formation can be seen, as shown in the image. The arrows indicate the upregulation and downregulation of genes during nitrogen and nutrition stress.

In *Nostoc*, PatS (a 13- to 17-aa-long protein with ID BAB74000.1) and HetN contain a conserved pentapeptide motif, RGSGR, essential for binding to HetR and eventually to turning it off (Xu et al., 2020). HetN and PatS regulate the location of heterocyst formation and prevent contiguous heterocyst formation (Corrales-Guerrero et al., 2014). In PCC 7120, several other pattern-forming proteins, such as HetZ and PatU, are involved in preventing multiple contiguous heterocyst formations (Zhang et al., 2007). However, we found that PatS is missing in UU774, PCC 9339, and NIES-3754. Since NIES-3754 and PCC9339 are near-complete genomes and share extensive synteny with UU774, the missing PatS is not likely due to the incomplete assembly of UU774. Our BLAST analysis of the PatS protein with other Hapalosiphonaceae members suggests that, although the N terminal of PatS matches with several members of this family, the “ERGS” motif matches with only few *Fisherella thermalis* species and, conspicuously, there are no matches with UU774, PCC9339, and NIES-3754. Interestingly, the HetN orthologs in UU774, NIES-3754, and PCC 9339 (BLD44_016650, WP_017313163, and BAU07447 respectively), lack the crucial DNA binding motif “RGSGR” (Xu et al., 2020), which is replaced by “QGNGH” (Supplementary Figure 11C). Surprisingly, in UU774, irrespective of nitrogen availability, hetN remains upregulated. Since hetN is responsible for heterocyst pattern formation, it could be anticipated that heterocysts should be formed at regular intervals. On the contrary, a microscopic study reveals that, under N– conditions, heterocysts are formed without a strict pattern and sometimes contiguously (Supplementary Figure 10B(ii)). The presence of an “H” residue in place of an “R” residue in the last residue of the motif QGNGH possibly alters the binding properties of this motif (Supplementary Figure 11D). HetF and PatA influence heterocyst development, presumably by acting downstream of HetR. It is believed that PatA attenuates the inhibitory roles of HetN and PatS (Orozco et al., 2006). In UU774, both HetF and PatA remain unperturbed during nitrogen stress and nutrient stress. Since PatS is missing and HetN lacks the functional binding motif, the expression of hetF and patA that acts upon HetN and PatS remains unchanged (Figure 5).

Recent studies indicate that HetL provides immunity to HetR and hence promotes heterocyst differentiation, even when PatS is active (Xu et al., 2020). In UU774, all the genes interacting with HetN and PatS remain unperturbed including HetZ, PatU, and HetL homologs (Table 2; Supplementary Table 4: Master). Since HetR is upregulated even in later time points, it is intriguing to see that proteins interacting with HetR inhibitors are unperturbed during later time points. HetZ and PatU are present as single-copy orthologs in all the four species under study. However, the PatU of PCC 7120 (BAB77625) has undergone a frameshift leading to significant changes in the N terminus region of the protein sequence (Supplementary Figure 11B). Therefore, it can be speculated that there is an altered heterocyst differentiation mechanism in place for UU774.

#### Absence of External Nitrogen During Starvation Has Adaptive Advantages Over the Presence of Nitrogen

In the presence of an external source of nitrogen, Heterocystous organisms take nitrogen from cells using a transport mechanism to carry out nitrogen assimilation. In UU774, a nitrate/nitrite transporter (BLD44_010685) and a nitrate reductase (BLD44_010690) are downregulated in starvation conditions but are unchanged during nitrogen stress. During nutrient stress, the unavailability of nitrate or nitrite in the external milieu leads to their downregulation. Interestingly, both of the genes are present in the genome as a single copy. The heterocyst differentiation protein HepA (BLD44_011825), Crp/Fnr-type global transcriptional regulator gene (BLD44_009930), and FeMo cofactor biosynthesis protein NifB (BLD44_004770) are significantly upregulated in the N– condition as expected. Global transcription regulators have multiple roles in bacteria and are implicated in stress response. Some of them directly respond to nitrogen stress (Zhou et al., 2012). In the case of PCC 7120, nitrogen starvation leads to unchanged expression of genes involved in nitrate reductase, nitrogen assimilation, and exopolysaccharide synthesis, but there is an increased expression in heterocyst differentiation and nitrogen regulator genes (Wei et al., 1994).

A conserved single copy nitrogenase-stabilizing/protective protein NifW2 (BLD44_019850) shows significant downregulation in the N+ condition. This may have an adverse effect on the structural integrity of nitrogenase leading to a reduced ability to fix nitrogen. Increased glutamine synthetase and reduced nitrate reductase activities were reported in non-heterocystous marine cyanobacteria *Oscillatoria willei* under nitrogen stress (Kumar Saha et al., 2003).

A magnesium transporter mgtE (BLD44_013840) is significantly downregulated in UU774 during nutrient stress but is upregulated in the N– condition, implying that the activity of magnesium transportation is reduced in prolonged exposure to nitrogen than in absence of it. This implies that the N– condition acts favorably for the uptake of ions regardless of the availability of nutrition. For photosynthetic electron transfer in cyanobacteria, iron, manganese, magnesium, and copper are some important cofactors. Uptake, utilization, and storage of these minerals are thus required to maintain metal homeostasis in cyanobacteria (Shcolnick and Keren, 2006).

Multidrug resistance ABC transporters (BLD44_013280) were significantly downregulated in the N+ condition (Supplementary Table 4: Master). A two-component signaling system (TCS), commonly known as bacterial IQ (Galperin, 2005), is considered a significant mode of signaling predominantly present in bacteria (Wuichet et al., 2010). The KEGG-KAAS analysis in UU774 indicates that there are 35 response regulators, 30 histidine kinases, and 2 putative two-component membrane permease complex subunits of a two-component signaling system, making them the second most abundant class (Canova et al., 2014) (Supplementary Table 7).

Only one copy of sensor histidine kinase TmoS (BLD44_026135) was upregulated in the N– condition, but it remained unperturbed during nutrient deprivation. TmoS/TmoT regulates the toluene-4-monooxygenase pathway in *Pseudomonas putida* (Silva-Jimenez et al., 2012). The toluene degradation pathway mediated by Tmo family genes is a known xenobiotic process. The Upregulation of TmoS/TmoT during nitrogen stress could be a possible indicator of a favorable response for the organism. During nutrient stress, VraR (BLD44_006700) is significantly upregulated but stays unperturbed during the N+ condition. The VraR regulation is linked with vancomycin antibiotic resistance (Canova et al., 2014). Therefore, it can be concluded that, during the N+ condition, protective mechanisms are severely compromised.

### Photosynthetic Efficiency Decreased in the Presence of Exogenous Nitrogen

UU774 exhibits increased expression of PSII proteins, including PsbA_D1 (BLD44_021265, BLD44_026620), Ferredoxin3 (BLD44_010210), and Ribulose bisphosphate carboxylase small chain (BLD44_008210), in the N– condition, suggesting increased photosynthesis. Additionally, there is increased Glucose/mannose transporter GlcP (BLD44_017550) activity. The carbonic anhydrase (CA) gene (BLD44_006795), responsible for the carbon concentrating mechanism, is upregulated during nitrogen depletion. An ortholog of this gene is missing both in NIES-3754 and PCC 7120 and is present only in PCC 9339 (WP_017307389.1). In many cyanobacteria, the CA gene is missing altogether (Badger and Price, 2003). The upregulation of photosynthetic apparatus in the N– condition probably gives an indication that there are some positive regulatory effects on overall photosynthetic machinery when their nitrogen-fixing mechanism is active. However, more experiments are needed to confirm this.

During nitrogen step down, there is reduced expression of storage product biosynthetic genes. For example, long-chain-fatty-acid-CoA ligase FadD15 (BLD44_028075) and glycogen synthase (BLD44_018145) genes are downregulated in the absence of nitrogen and remain unperturbed during nutrition stress. Recent studies on *Synechococcus elongatus* PCC 7942 suggest that degradation of storage products such as glycogen is the first step toward stimulation of photosynthesis (Shinde et al., 2020). Active photosynthesis is a stage where an organism tends to utilize reduced storage products; hence, there could be reduced biosynthesis of glycogen, as seen in non-diazotrophs (Neumann et al., 2021). Therefore, it could possibly be speculated that during nitrogen step down, UU774 remains photosynthetically more active than when nitrogen is supplied.

### Compelling Evidence Suggests Long-Term Exposure to Nitrogen Leads to Multiple Adverse Effects on UU774

Expression of vital enzymes such as alkaline phosphatase synthesis transcriptional regulator SphR (BLD44_003370), nitrogenase-stabilizing/protective protein NifW2 (BLD44_019850), phosphate-binding protein PstS (BLD44_028190), biotin, and cofactor biosynthesis (BLD44_000510) are reduced in the presence of nitrogen (Figure 4). SphR plays a significant role in the production of alkaline phosphatase in response to phosphate limitation, and the reduced expression of SphR could imply the reduced ability to convert phosphate into a bio-available form. We discuss major genes perturbed during nitrogen stress below.

#### Co-Transcribed Clusters of Mobility Response Regulators Downregulated in the Presence of External Nitrogen

**Figure 6:**
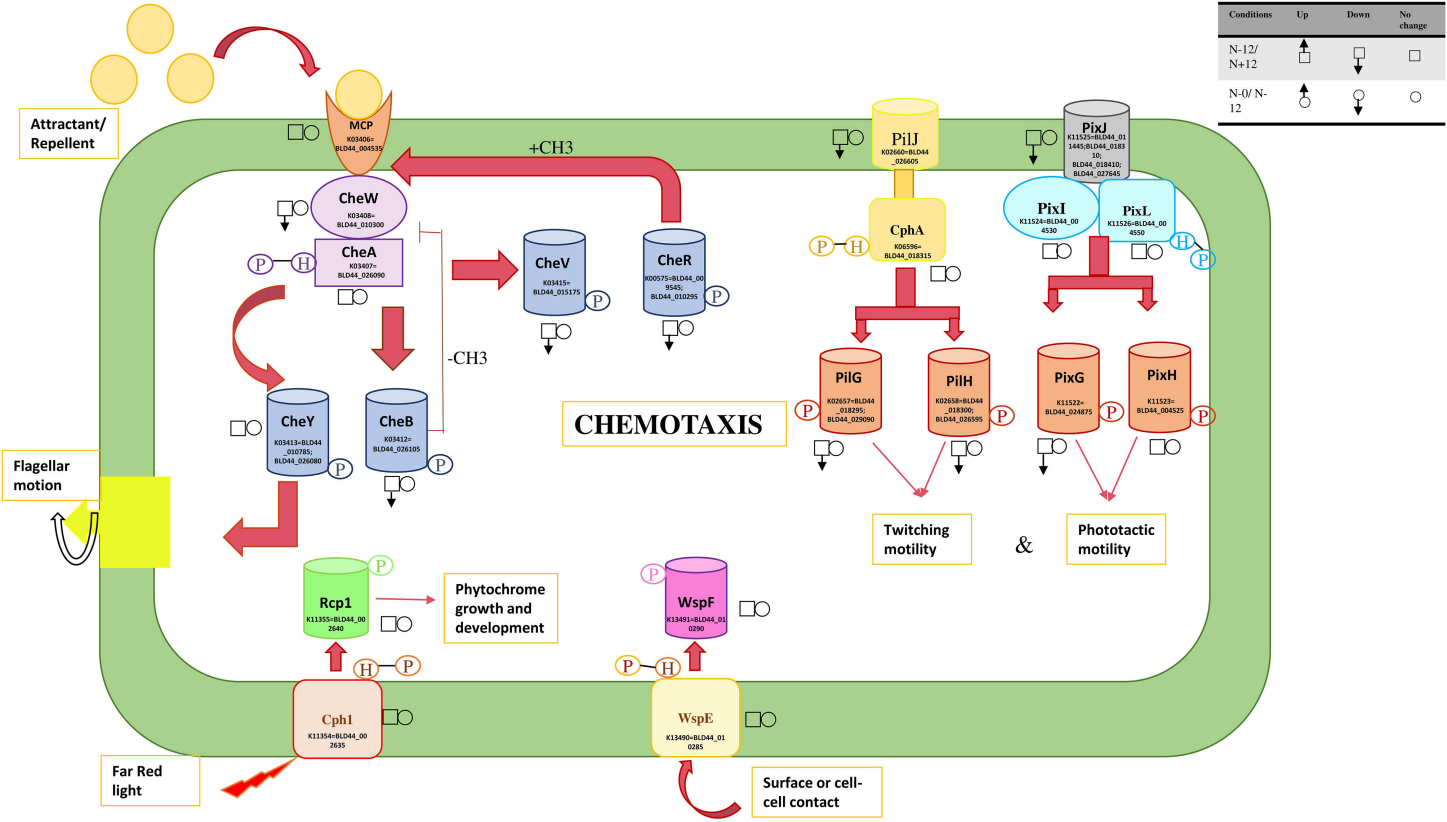
Response regulators involved in the movement and their expression levels under nitrogen and nutrition stress. The square boxes represent expression during nitrogen stress in N– condition on the 12th day as the reference and are compared with the N+ condition on the 12th day. For nutrition stress, circles are used with up and down arrows to represent upregulation and downregulation. The reference here is the 0th day under culture in N– conditions and is compared with the 12th day in culture under N– conditions. The signal transducers and response regulators are downregulated during the N+ condition, leading to substantially reduced mobility.

A mobility gene cluster (BLD44_011130 through BLD44_011145), BLD44_026465, BLD44_023445, BLD44_023450, BLD44_005625, BLD44_011135, and BLD44_027030 are downregulated during the N+ condition, indicating an overall decrease in movement (Figure 6). BLD44_011135 and BLD44_027030, which code for methyl-accepting chemotaxis protein McpB and Fimbrial protein, respectively, show a low level of downregulation, but all other motility-related genes are unperturbed during the starvation condition. A plasmid-derived cluster in scaffold_13 containing transporters and a cupin-like domain (BLD44_013115 through BLD44_013275), collinear with a plasmid of PCC 9339, JH992890, is significantly upregulated during the starvation condition and helps survive stress condition (Supplementary Table 5: all collinearity ka/ks' file).

Several signal sensors and their cognate response regulators are downregulated during the N+ condition. Signal sensor CheW (BLD44_010300; BLD44_011140) and its corresponding response regulator CheV (BLD44_015175); twitching motility pairs of signal sensors and response regulators, PilJ (BLD44_026605) and PilH (BLD44_026505); response regulator pairs induced in response to light, such as PixJ (BLD44_011445; BLD44_018310; BLD44_018410; and BLD44_027645) and PixG (BLD44_024875), are downregulated during the N+ condition. We measured the movement and found that UU774 shows increased mobility in the N– condition (Supplementary Movie S1). The filaments showing no movement during prolonged nitrogen exposure only corroborates this fact.

#### Reduced Cell Division and Growth

A chromosomal replication gene, dnaA (BLD44_014095:IPR001957); the SepF protein responsible for septa formation (BLD44_015735); a WalR-K signal transduction protein (BLD44_011145); a chromosome partition protein Smc (BLD44_026585); Murein hydrolase activator NlpD, required for daughter cell separation (BLD44_028525); histidine protein kinase DivJ required for the regulation of cell division and differentiation (BLD44_018165); cytoskeleton protein RodZ (BLD44_017275); and proteins involved in RNA polymerase sigma-F factor (BLD44_014205, BLD44_018865) are downregulated during the N+ condition. Surprisingly, nutrition depletion does not affect the expression of these genes (Figure 3).

RNA polymerase Sigma-F factors (BLD44_014205 and BLD44_018865) are present in single copies, and these are conserved across the 4 species. These are structurally dissimilar with bacteria as they lack gyrB genes in the cluster downstream of dnaA (Skarstad and Boye, 1994). The SepF protein recruits tubulin in forming a Z ring that is essential for the assembly of cell division proteins (Duman et al., 2013). The WalR-K signaling system is a conserved system coordinating cell wall metabolism with growth in bacteria (Dobihal et al., 2019). Strong downregulation of WalR-K refers to a lack of coordination between cell growth and synthesis of cell wall components during the N+ condition (Takada et al., 2018). We found that the N+ filaments are shorter than the N– filaments (Figure 4). Contrastingly, in PCC 7120, the presence of combined nitrogen (either nitrate or ammonia) leads to the formation of long filaments (Kumar et al., 2010).

#### Increased Expression of Sporulation-Related Genes and Reduced Biofilm Production in the Presence of Nitrogen

Sporulation initiation inhibitor protein Soj (BLD44_021615 and BLD44_014135), S8 and S53 subtilisin (BLD44_029995), and putative glycosyltransferase EpsE (BLD44_018425) responsible for biofilm production are downregulated in the N+ condition, signifying an impending sporulation-like condition. Soj (BLD44_021615) does not have an ortholog in its closest relatives PCC 9339 and NIES-3754 but has an ortholog in PCC 7120 (BAB73070). Subtilisins are serine endopeptidases often playing important roles in defense and repair mechanisms and stress responses (Silva et al., 2002). Reduced expression of these genes suggests a reduced ability to ward-off danger.

Biofilm production is a defense mechanism for prokaryotic organisms by which they are protected against drugs and other external agents. For efficient biofilm formation, proteins containing GG motifs need to be cleaved by a cysteine protease (Parnasa et al., 2016). A constitutive downregulation of peptidases in the presence of nitrogen reduces the biofilm production ability of UU774, which is evident in our microscopic studies (Figure 4). A recent study demonstrates that exogenous nitrogen and phosphorus fertilizers induce a pathogenic response that needs to be studied in the present context (Lekberg et al., 2021).

### Heat Resistance Possibly Attributed to the Presence and Constitutive Expression of Abundant Peptidases

Heat shock treatment in UU774 has resulted in the least number of differentially expressed genes, suggesting a constitutive model of gene expression for all major heat-resistant genes including a heat-inducible transcription repressor HrcA (BLD44_004490).

During 6 h of heat shock, significant upregulation of an M28 family metallo-peptidase (BLD44_000095) is most notable (Supplementary Table 4: Master; Supplementary Figure 4). Peptidases are responsible for the degradation of proteins. Under heat shock conditions, many proteins may be misfolded, damaged, and mislocalized (Ali and Baek, 2020). We also report the presence of 3 times more number of peptidases in hot spring species compared to non-hot spring PCC 7120 species (UU774: 78, PCC 9339: 89, NIES-3754: 80, and PCC 7120: 30). Other highly perturbed proteins include, naphthalene 1, 2-dioxygenase (BLD44_006725), a singleton present only in UU774 that is involved in the degradation of naphthalene, and an NYN domain containing a protein with an unknown function (BLD44_017675). These proteins belong to the family of nucleases and are probably involved in the degradation of abnormal proteins (Anantharaman and Aravind, 2006). BLD44_017675 is present in single copies in PCC 9339 and NIES-3754, and its ortholog is missing in PCC 7120 (Supplementary Table 4: Ortholog all clusters; Supplementary Figure 12). A disorder prediction protein (BLD44_005905) is significantly upregulated, implying its possible role in the protein degradation pathway. This protein is a singleton and is unique to UU774. N-acetylmannosamine-6-phosphate 2-epimerase (BLD44_018895) is perturbed during heat stress and has an ortholog in PCC 9339 but is absent in PCC 7120 and NIES-3754. This protein is reportedly implicated in heat and oxidative stress (Dijkstra et al., 2014). A gene coding for HSP83_3_UTR (BLD44_008410) and exclusively present in UU774 is significantly perturbed. HSP83_3_UTR, in *Leishmania*, controls the translation of Hsp83 in a temperature-controlled manner (David et al., 2010). Furthermore, several small hypothetical proteins are perturbed including a dihydrofolate reductase (KNPGNMGK_04634).

A study on cyanobacteria coping with heat stress exclusively discussed Hsp proteins (Rajaram et al., 2014). We conclude that nucleases and peptidases play a significant role in heat-induced damage to the organism.

## Conclusion

*Mastigocladus laminosus* UU774, a hot spring strain, possibly combats heat stress by constitutive expression of heat-resistant genes and maintaining a high copy number of peptidases that are responsible for the removal of malformed proteins. During prolonged starvation, UU774 copes well without nitrogen than in the presence of it. During prolonged exposure to nitrogen, cells undergo severe stress, including reduced ability to photosynthesize, and permeate nutrients, protecting themselves from impeding pathogens leading to the initiation of sporulation as is evident in gene expression studies. In the absence of nitrogen, on the other hand, the organism remains energetically viable by optimizing the photosynthetic apparatus, secreting toxins and effectors, producing biofilms, and remaining phototactically motile, indicating an indirect role of nitrogen fixation on the overall wellbeing. Heterocyst spacing is not orchestrated well in hot spring sp. because of the absence of heterocyst disrupter PatS and loss of functional motif RGSGR in its cognate HetN. The presence of the highest number of orthologs between UU774 and PCC 9339 indicates that these species originated together before their geographic separation and speciation. The preservation of certain mutations leading to gene death between UU774 and PCC 9339 is intriguing. This study has a significant global implication on long-term exogenous nitrogen exposure to fields leading to reduced functionalities of naturally harboring cyanobacteria. Further wet-lab studies are required to validate the hypothesis drawn from the genome analysis.

### Limitations of the Study

Our hypothesis is purely based on genome data and gene expression patterns as observed by RNAseq and qRT-PCR. This study lacks proteomics analysis. Furthermore, we have only taken 2 time points and 2 experimental conditions, that is, 0th day and 12th day, and the effect of starvation with or without nitrogen. The time point is based on our pilot experiments and indicates that there is a metabolic switch on the 12th day. Time points in between could have unfolded the gradual changes in expression levels leading to loss of fitness.

## Data Availability Statement

UU774 was deposited at ICAR-National Bureau of Agriculturally Important Microorganisms having accession number TC-01457. Raw reads of UU774 were submitted to NCBI (SRS3624828). The genome assembly has accession number MNPM02000000. The Bioproject ID is PRJNA350610 and Biosample ID is SAMN05942614. Prokka annotation is available at https://doi.org/10.6084/m9.figshare.12941690.v1. RNAseq pipeline available at https://github.com/computational-genomics-lab/cyanobacteriaRNAseqPipeline. All the scripts used for data analysis are available at https://github.com/computational-genomics-lab/UU774Accessory.

## Author Contributions

ST conceived the project. MM and ST designed the project. ST, MM, and AG analyzed the results. AG, SG, ANS, SD, AP, and SB helped in analyzing the data. ST, MM, and AG wrote the manuscript. All authors contributed to the article and approved the submitted version.

## Funding

ST would like to acknowledge DBT, Ramalingaswamy fellowship, and CSIR for supporting this study. MM would like to acknowledge CSIR for the fellowship. ANS was funded by the CSIR pool scientist scheme. SG and AG were funded by the DBT fellowship. SD was supported by the ICMR fellowship. AP was supported by the UGC fellowship.

## Conflict of Interest

The authors declare that the research was conducted in the absence of any commercial or financial relationships that could be construed as a potential conflict of interest. The reviewer KP declared a shared affiliation with the author SA to the handling editor at the time of review.

## Publisher's Note

All claims expressed in this article are solely those of the authors and do not necessarily represent those of their affiliated organizations, or those of the publisher, the editors and the reviewers. Any product that may be evaluated in this article, or claim that may be made by its manufacturer, is not guaranteed or endorsed by the publisher.

## Supplementary Material

Supplementary Figure 1 AlphaFold v2.1.0 predicted 3D models of HetN protein from **(A)** *Nostoc* sp. PCC 7120, **(B)** *Fischerella* sp. NIES-3754, **(C)** *Mastigocladus laminosus* UU774, and **(D)** *Fischerella* sp. PCC 9339, and their corresponding predicted local distance difference test (pLDDT) and predicted aligned error (PAE) plots.

Supplementary Figure 2 Annotated files of the UU774 genome and its plasmids were plotted using the standalone version of DNAPlotter. The outermost ring is the coding sequences in the plus strand and the second ring is the coding features in the minus strand. Ring 3 is for r-RNA genes and ring 4 contains t-RNA genes and other genomic features. The innermost two rings are the GC skew in the sense and anti-sense strands and the GC contents, respectively. The GC skew is calculated as [(G-C)/(G+C)].

Supplementary Figure 3 A boxplot illustrating inter-genic distance of the four species. Plotting was performed using the R script (submitted to GitHub page). Intergenic distance *vs*. gene length plot for **(A)** UU774, **(B)** PCC 9339, **(C)** NIES-3754, and **(D)** PCC 7120.

Supplementary Figure 4 **(A)** Average nucleotide identity and **(B)** Tetranucleotide values were computed between the scaffolds of UU774 to determine the degree of relatedness among them using the pyani package. The data were plotted using an R script (deposited in GitHub page). Plasflow-identified plasmids and scaffolds, cryptic scaffold_38, and contaminated scaffold_20 were highlighted in red, yellow, and green, respectively.

Supplementary Figure 5 Validation of RNAseq transcripts by qRT-PCR. Each of the experimental conditions was performed with three biological replicates. For each of the biological replicates, three technical replicates were used. The Log2 fold change values obtained from qRT-PCR and RNAseq analysis for a single gene are placed sidewise. **(A)** Representative image showing the cluster of genes from scaffold_38 is depicted. **(B)** qRT-PCR validation of transcripts under nutrient stress. **(C)** qRT-PCR validation of transcripts under heat shock conditions.

Supplementary Figure 6 Pseudo-gene transglutaminase BLD44_015595, which is present in HetL/PatU and the glutamate racemase (implicated in peptidoglycan biosynthesis) cluster in scaffold_14, is present in its functional version in the Japanese strain NIES-3754 and *Nostoc* sp. PCC 7120. This gene is present as a pseudo-gene in both UU774 and PCC 9339, with the introduction of a stop codon in the 13th position, where a TGG is converted into a TAG. The phylogenetic tree of this gene indicates that the gene has gained a function later in the Japanese species.

Supplementary Figure 7 **(A)** ANI identity among the 45 Hapalosiphonaceae species studied for comparison purposes where UU774 was used as a reference (highlighted in red). PCC 9339 and NIES-3754, highlighted in yellow and green, respectively, are used for comparison. **(B)** Presence of mobile genetic elements in 45 members of the Hapalosiphonaceae family. Crisper finder, ISsaga, and Phaster were used to calculate CRISPR CAS elements, IS sequences, and Phage elements, respectively.

Supplementary Figure 8 A Synteny plot between scaffold_4 of UU774 with collinear fragments in PCC 9339 and NIES-3754. BLD44_006515 codes for an IS5 element that is pseudogenized. The fragments that follow BLD44_006515 are functional genes, but the synteny with PCC 9339 is lost in that region. Genes “WP_017308697.1” and “WP_051040561.1” belong to plasmid JH992893.1 and have lost synteny between them. The genes from BLD44_006670 to BLD44_006810 do not have synteny with PCC 9339 but have some genes from NIES-3754 as orthologs and lack conserved synteny between them. The same can be said about the genes BLD44_006860 and BLD44_006970.

Supplementary Figure 9 **(A)** A Synteny plot showing large scale genome re-arrangements among hot spring species UU774, PCC9 339, and NIES-3754. The upper panel contains synteny between UU774 and NIES-3754 and the lower panel contains UU774 with PCC 9339 and UU774 scaffolds that are represented in the middle. **(B)** A circular synteny plot among UU774, PCC 9339, and NIES-3754. **(C)** A mummerplot between UU774 and PCC 9339. **(D)** A mummerplot between UU774 and NIES-3754.

Supplementary Figure 10 **(A)** (i) Boxplot depicting a number of vegetative cells in cultures grown continuously under N+ and N– conditions for 12 days. (ii) Microscopic images of UU774 cultures grown under N+12 and N-12 conditions, where N– grown cultures have longer filament lengths while N+ cultures form clumps and have shorter filament lengths. **(B)** (i) Number of heterocysts present in a single filament in cultures grown continuously under N+ and N– conditions for 12 days. For each of these, 3 biological replicates were taken. From each replicate, 5 slides were made, and from each slide, 5 observations were taken. (ii) Microscopic image of N– grown filament with a contiguous heterocyst suggests disruption in heterocyst pattern formation of UU774 (red arrow).

Supplementary Figure 11 **(A)** A multiple sequence alignment of HetP genes in UU774, PCC 9339, NIES-3754, and PCC 7120. While all the hot spring species contain a single copy of the genes, PCC 7120 contains 2 copies of this gene. The genes show a strong negative purifying selection. Between the two paralogues of HetP in PCC 7120, BAB74517 is more similar to the hot spring strains. **(B)** Multiple protein sequence alignment of PatU among the 4 species clearly shows that PCC 7120 has a frame shift leading to the substantial alteration in protein sequence at the N terminus. **(C)** Multiple protein sequence alignment among the HetN of UU774, PCC 9339, NIES-3754, and PCC 7120 indicates that the DNA-binding motif ERGSGR present in PCC 7120 is altered in all the 3 hot spring species. In UU774 and PCC 9339, it is changed into DQGNGH, whereas in NIES-3754, it is changed into ERNSGH, hence leading to the loss of binding affinity to DNA to carry out HetR transcription. **(D)** Superimposition of Alphafold v2.1.0-predicted HetN structure from UU774, PCC 9339, NIES-3754, and PCC 7120.

Supplementary Figure 12 **(A)** Table representing the shared orthologous gene clusters among the four species. The left pattern shows the species sharing the gene clusters; cluster count refers to the number of clusters shared between species and protein count is the total protein number in shared species. **(B)** Venn diagram showing the distribution of ortholog gene clusters among four species. **(C)** (i) Heatmap indicating the number of overlapping gene clusters between each pair of genomes (ii) Phylogenetic tree generated between UU774, PCC 9339, NIES-3754, and PCC 7120.

Supplementary Table 1 RT PCR primers and validation, heterocyst count, and filament length. Sheet 1. Gene IDs (both Genbank and Prokka), genomic location, and the primer sequences used for the qRT-PCR experiments. Sheet 2. Results of the qRT-PCR experiments. For each of the experimental conditions, three biological replicates were used to extract RNA. For qRT- PCR, three technical replicates for each of the biological replicates were used. Sheet 3. Raw counts of the number of vegetative cells in a single filament and the number of heterocysts in each filament in cultures grown continuously under N+ and N– conditions for 12 days. For each of the N+ and N– conditions, 3 biological replicates were taken. From each replicate, 5 slides were made, and from each slide, 5 observations were taken. The slides were observed under a light microscope at 100X while counting. Sheet 4. Raw counts of the length of single filaments (in μm) of cultures grown continuously under N+ and N– conditions for 12 days. For each of the N+ and N– conditions, 3 technical replicates were taken. From each replicate, 3 readings were made. The slides were observed under a light microscope at 20X while counting.

Supplementary Table 2 Intergenic distances between the genes were calculated using in-house Perl scripts (submitted to GitHub).

Supplementary Table 3 Predicted plasmids, genome islands, and phage elements. Sheet 1. PlasFlow prediction results for UU774. Sheet 2. PlasFlow prediction results for PCC 9339. Sheet 3. Genomic islands predicted from UU774. Sheet 4. Phaster-predicted phage elements from UU774.

Supplementary Table 4 Master expression file. Sheet 1. Differential expression analysis results using Kalisto are presented for genes with their IDs and location along with their log 2-fold expression change and *p*-values. Sheet 2. Raw normalized read count for genes using R. Sheet 3. Genome size and total coding length. Sheets 4–8. Individual treatments and their expression values. Sheet 9. Annotation gff file for UU774 using Prokka. Sheet 10. Annotation gff file by the PGAP annotation present on Genbank. Sheets 11–14: Curated short-listed genes having a certain level of expression for further downstream data analysis. Sheet 15. Annotated features other than CDSs.

Supplementary Table 5 Cluster data generated using OrthoVenn2. Singletons and unique clusters present in UU774 are depicted. Sheet 1. All the clusters found by Orthovenn2 analysis. Sheet 2. Clusters common to all the four species. Sheet 3. Clusters common to UU774 and PCC 7120. Sheet 4. Singletons present in UU774. Sheet 5. Unique clusters present in UU774. Sheet 6. First, blastP was run on protein sequences of UU774, PCC 9339, NIES-3754, and PCC 7120, with each other and within the same genome. The output results along with the genome feature files were passed on to MCscanX to calculate collinearity. The dN/dS ratio was calculated to identify genes undergoing positive and negative purifying selection. Sheet 7. Tandem duplicated genes of all the 4 analyzed species are presented using MCscanX.

Supplementary Table 6 Blastn search between the “functional group II intron reverse transcriptase/maturase” gene (BLD44_007940) and the PCC 9339 genome and its plasmids. The blast result points to the 404344-to-406081 region of plasmid JH992892.1 containing a frame-shift leading to a pseudo-gene. The gene ID is RS35460 (PCC 9339). This gene is absent in PCC 7120, NIES-3754, and other cyanobacteria. Only a rudimentary copy of this gene is present in NIES-4106 plasmid DNA.

Supplementary Table 7 The number of genes present in metabolic pathways present in the four species studied by KEGG/KAAS. The links are given to pathways present in UU774.

Supplementary Movie 1 Video showing motility of N– grown UU774 filaments. The video was captured on time-lapse mode under 100X using a light microscope.
