# Supplementary figures and images for: Genome Analysis Coupled With Transcriptomics Reveals the Reduced Fitness of a Hot Spring Cyanobacterium Mastigocladus laminosus UU774 Under Exogenous Nitrogen Supplement
Source: Front Microbiol. 2022 Jul 1;13:909289. doi: 10.3389/fmicb.2022.909289 (PMC9284123; doi:10.3389/fmicb.2022.909289)

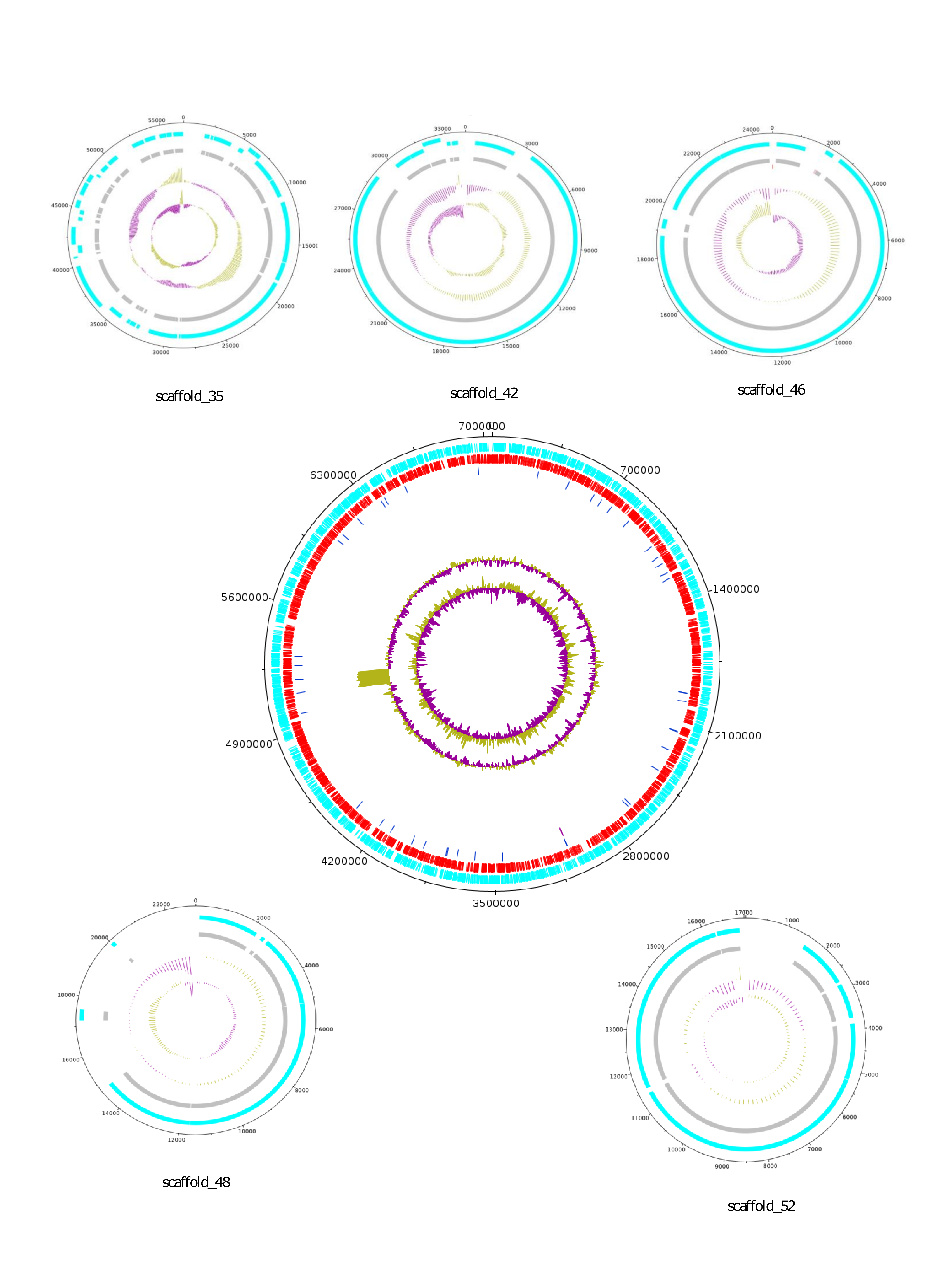

Supplement: Supplementary Figure 2 — Annotated files of the UU774 genome and its plasmids were plotted using the standalone version of DNAPlotter. The outermost ring is the coding sequences in the plus strand and the second ring is the coding features in the minus strand. Ring 3 is for r-RNA genes and ring 4 contains t-RNA genes and other genomic features. The innermost two rings are the GC skew in the sense and anti-sense strands and the GC contents, respectively. The GC skew is calculated as [(G-C)/(G+C)]. [file Image_2.TIFF]

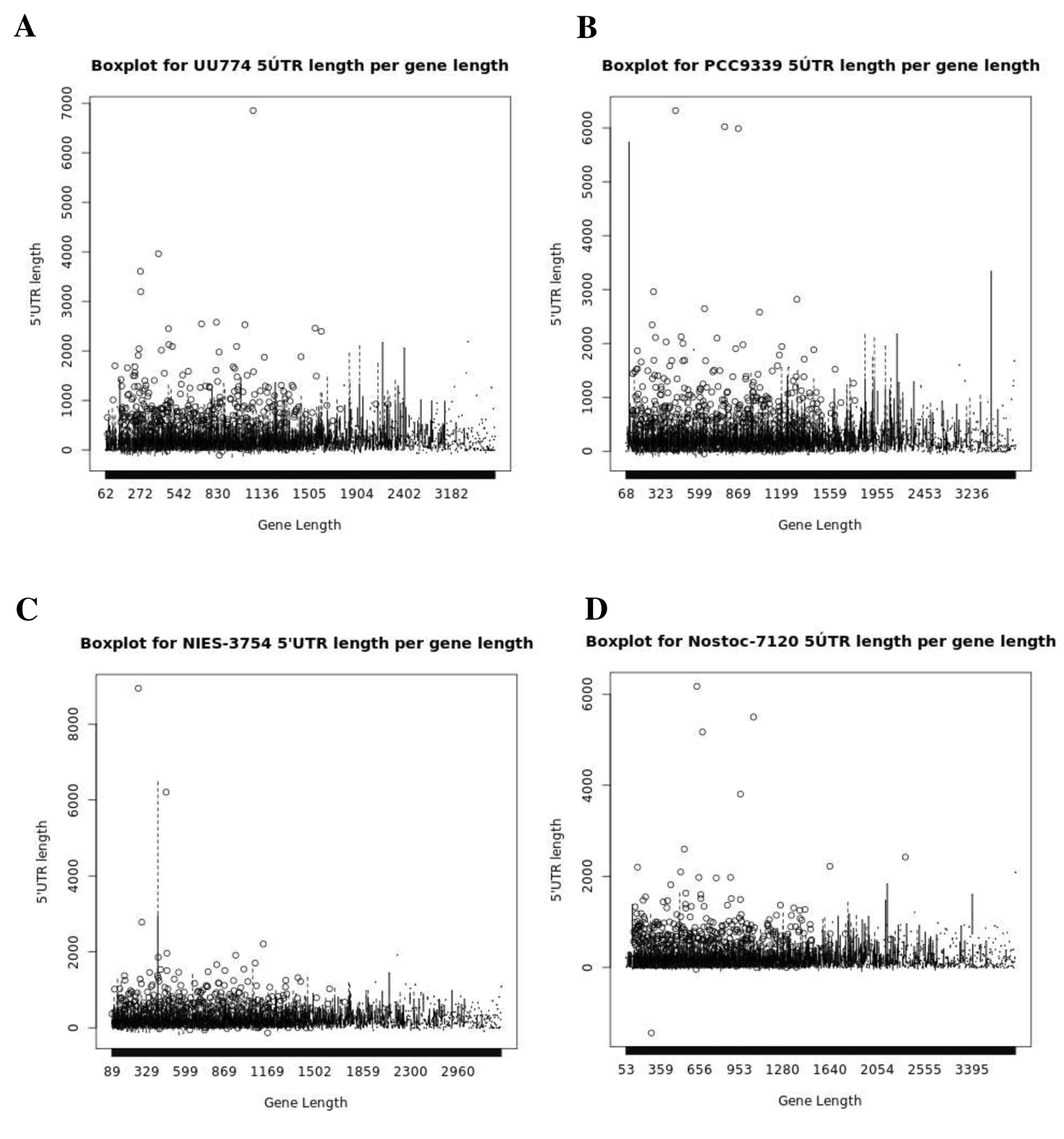

Supplement: Supplementary Figure 3 — A boxplot illustrating inter-genic distance of the four species. Plotting was performed using the R script (submitted to GitHub page). Intergenic distance vs. gene length plot for (A) UU774, (B) PCC 9339, (C) NIES-3754, and (D) PCC 7120. [file Image_3.TIF]

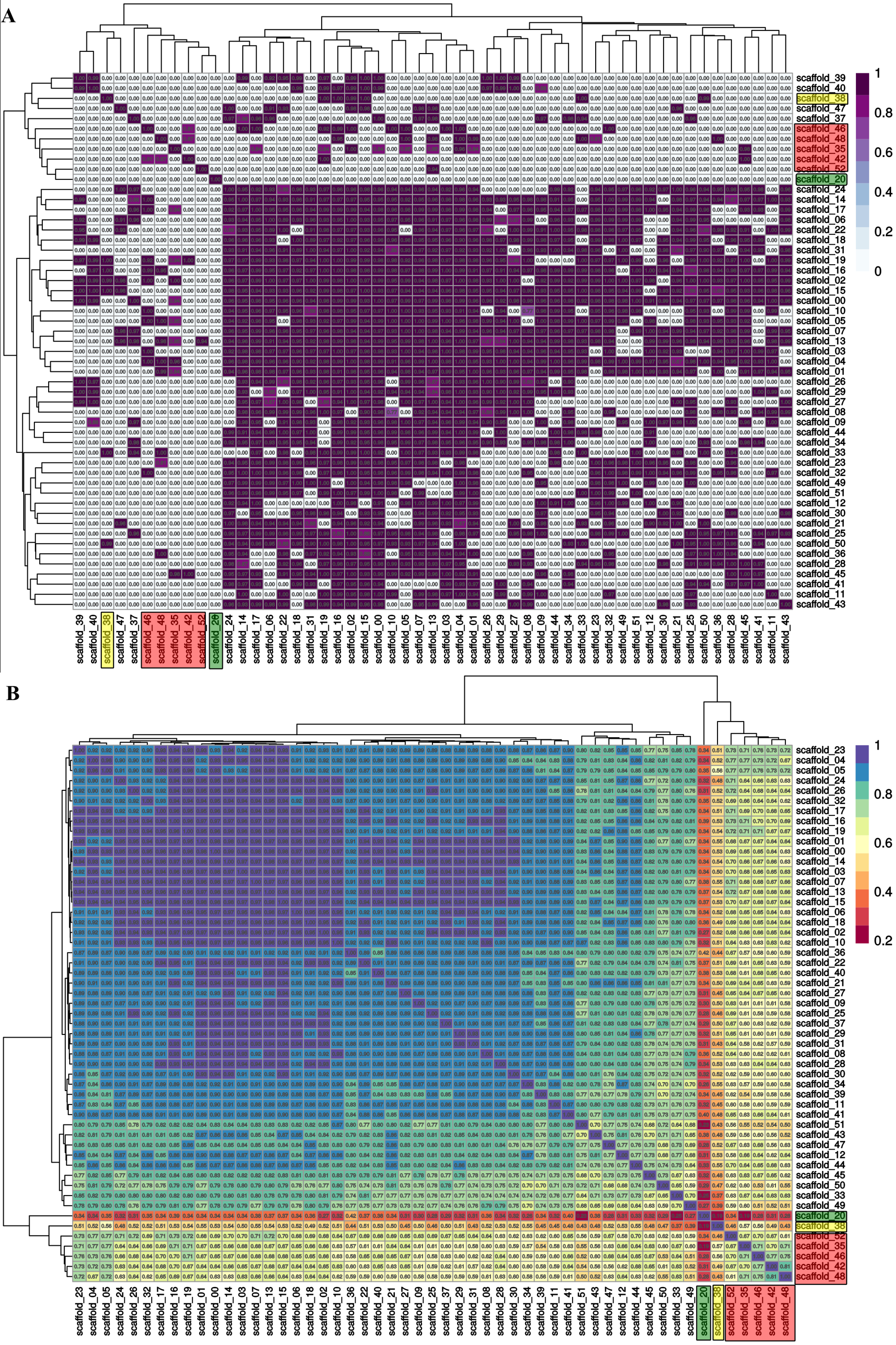

Supplement: Supplementary Figure 4 — (A) Average nucleotide identity and (B) Tetranucleotide values were computed between the scaffolds of UU774 to determine the degree of relatedness among them using the pyani package. The data were plotted using an R script (deposited in GitHub page). Plasflow-identified plasmids and scaffolds, cryptic scaffold_38, and contaminated scaffold_20 were highlighted in red, yellow, and green, respectively. [file Image_4.TIF]

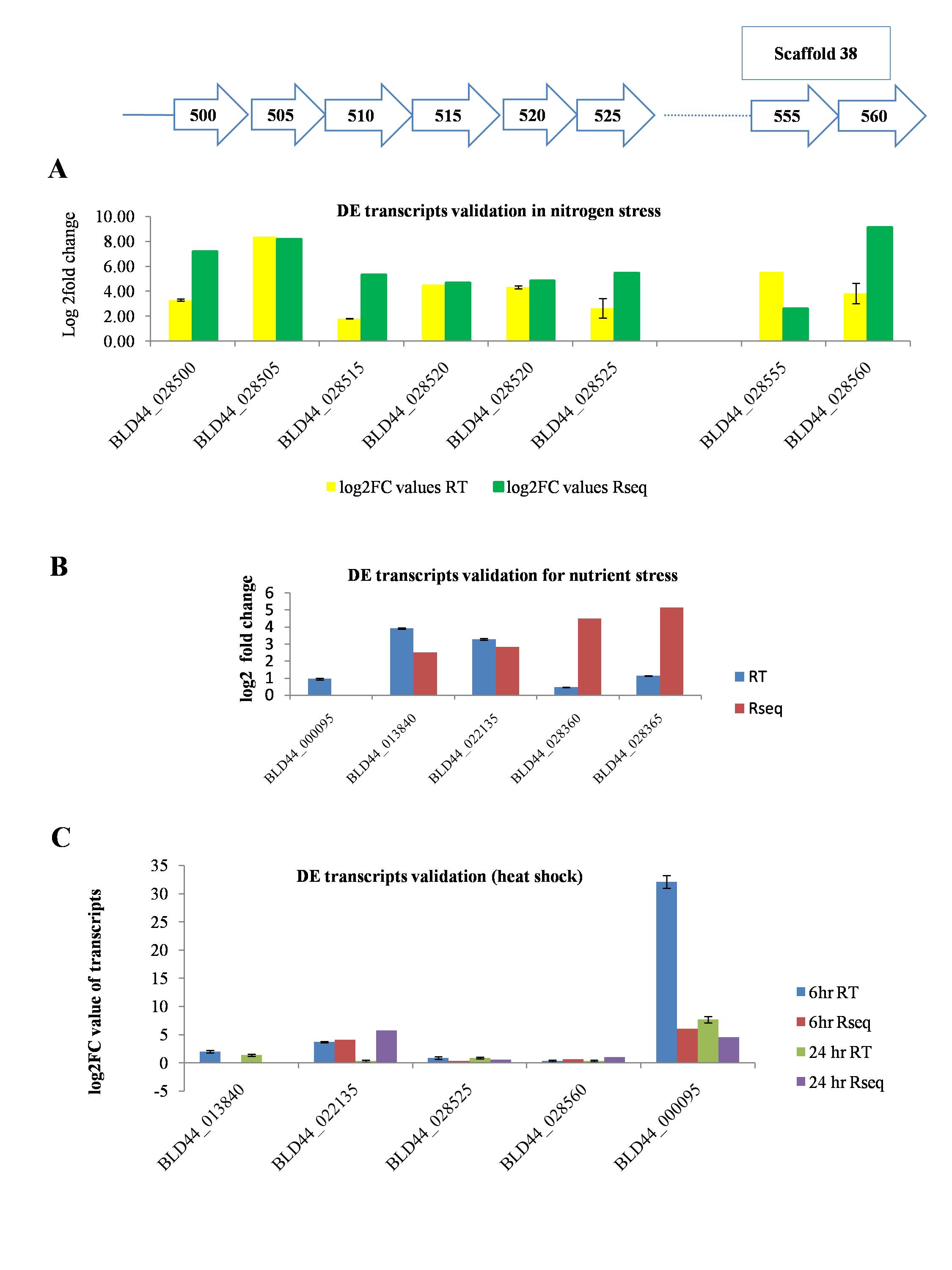

Supplement: Supplementary Figure 5 — Validation of RNAseq transcripts by qRT-PCR. Each of the experimental conditions was performed with three biological replicates. For each of the biological replicates, three technical replicates were used. The Log2 fold change values obtained from qRT-PCR and RNAseq analysis for a single gene are placed sidewise. (A) Representative image showing the cluster of genes from scaffold_38 is depicted. (B) qRT-PCR validation of transcripts under nutrient stress. (C) qRT-PCR validation of transcripts under heat shock conditions. [file Image_5.JPEG]

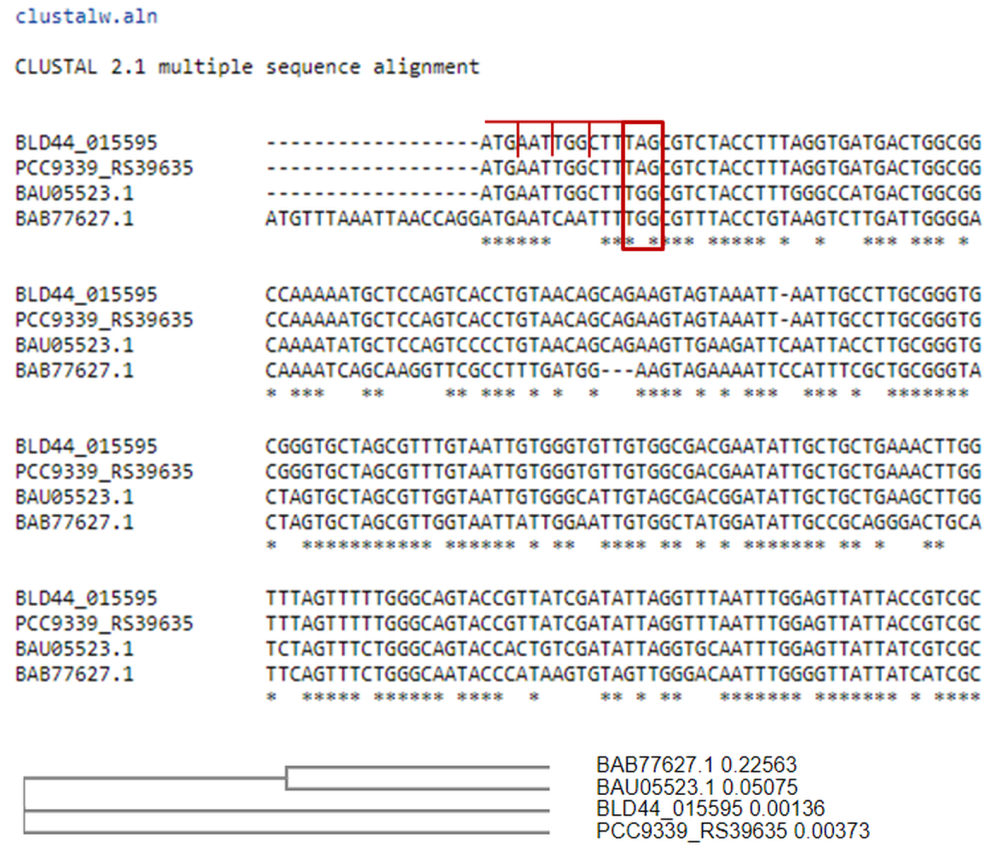

Supplement: Supplementary Figure 6 — Pseudo-gene transglutaminase BLD44_015595, which is present in HetL/PatU and the glutamate racemase (implicated in peptidoglycan biosynthesis) cluster in scaffold_14, is present in its functional version in the Japanese strain NIES-3754 and Nostoc sp. PCC 7120. This gene is present as a pseudo-gene in both UU774 and PCC 9339, with the introduction of a stop codon in the 13th position, where a TGG is converted into a TAG. The phylogenetic tree of this gene indicates that the gene has gained a function later in the Japanese species. [file Image_6.TIF]

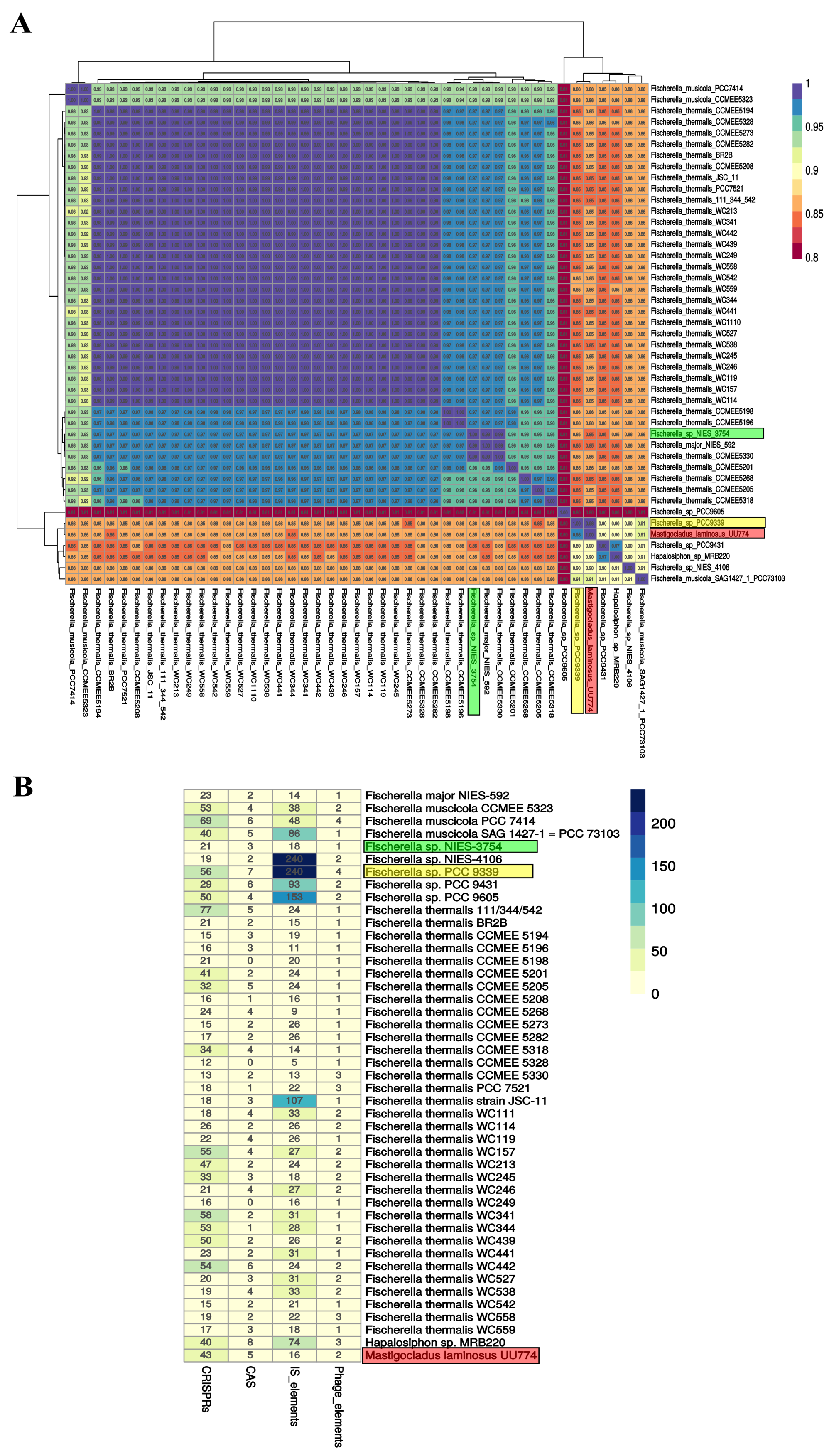

Supplement: Supplementary Figure 7 — (A) ANI identity among the 45 Hapalosiphonaceae species studied for comparison purposes where UU774 was used as a reference (highlighted in red). PCC 9339 and NIES-3754, highlighted in yellow and green, respectively, are used for comparison. (B) Presence of mobile genetic elements in 45 members of the Hapalosiphonaceae family. Crisper finder, ISsaga, and Phaster were used to calculate CRISPR CAS elements, IS sequences, and Phage elements, respectively. [file Image_7.TIF]

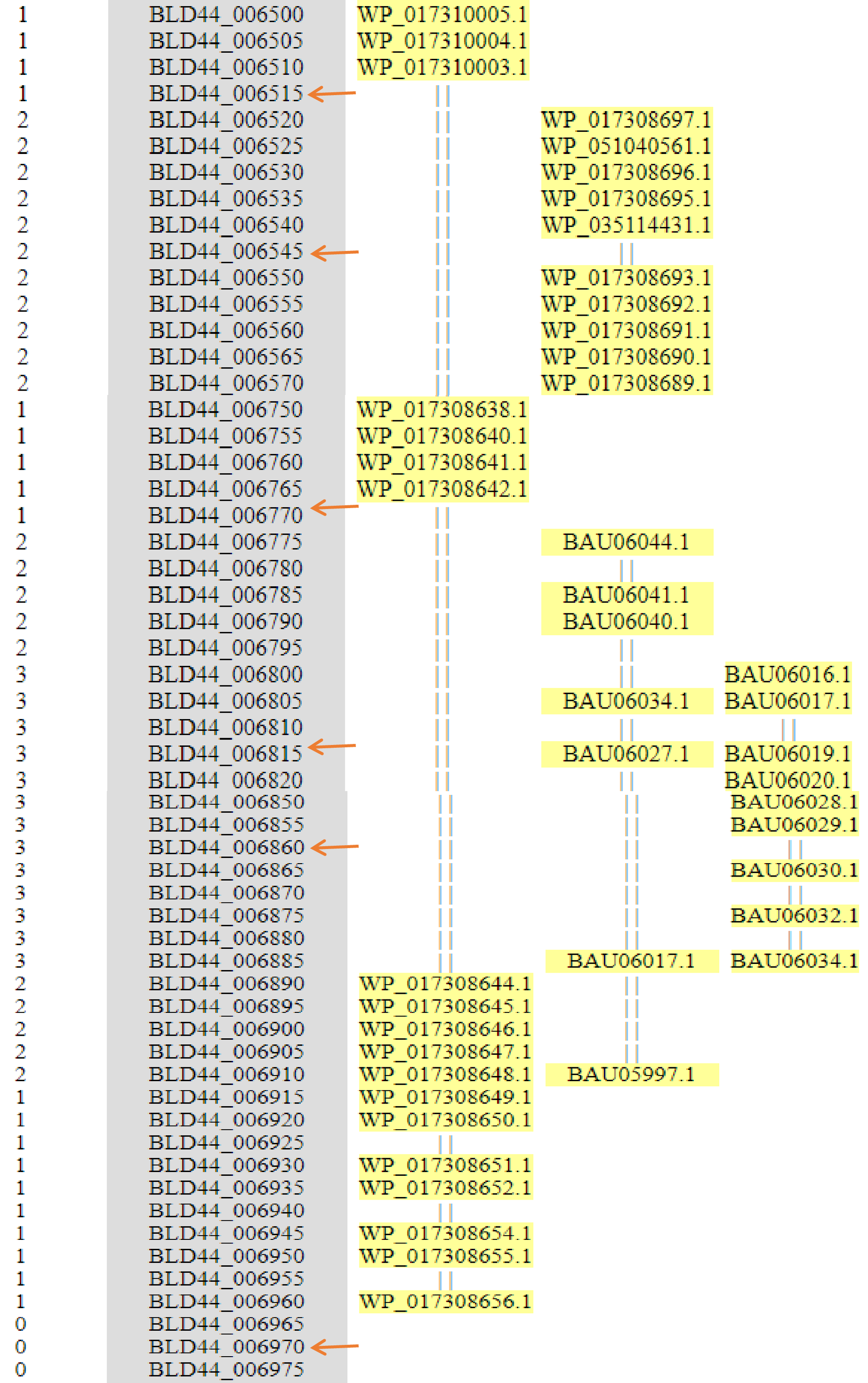

Supplement: Supplementary Figure 8 — A Synteny plot between scaffold_4 of UU774 with collinear fragments in PCC 9339 and NIES-3754. BLD44_006515 codes for an IS5 element that is pseudogenized. The fragments that follow BLD44_006515 are functional genes, but the synteny with PCC 9339 is lost in that region. Genes “WP_017308697.1” and “WP_051040561.1” belong to plasmid JH992893.1 and have lost synteny between them. The genes from BLD44_006670 to BLD44_006810 do not have synteny with PCC 9339 but have some genes from NIES-3754 as orthologs and lack conserved synteny between them. The same can be said about the genes BLD44_006860 and BLD44_006970. [file Image_8.TIF]

**Alignment of UU774 functional group II intron reverse transcriptase/maturase” gene (BLD44_007940) with PCC 9339**


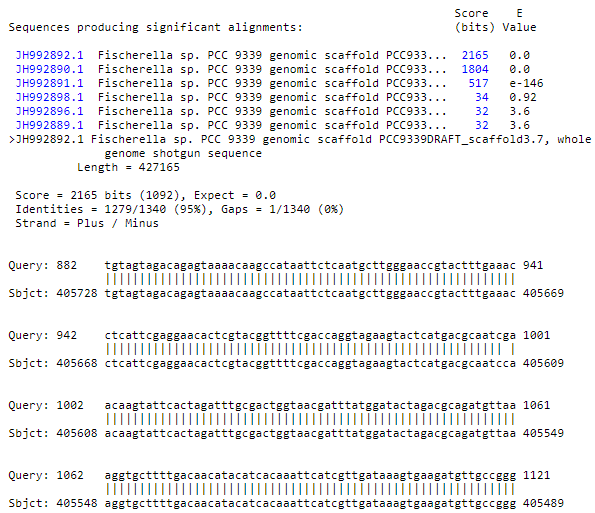


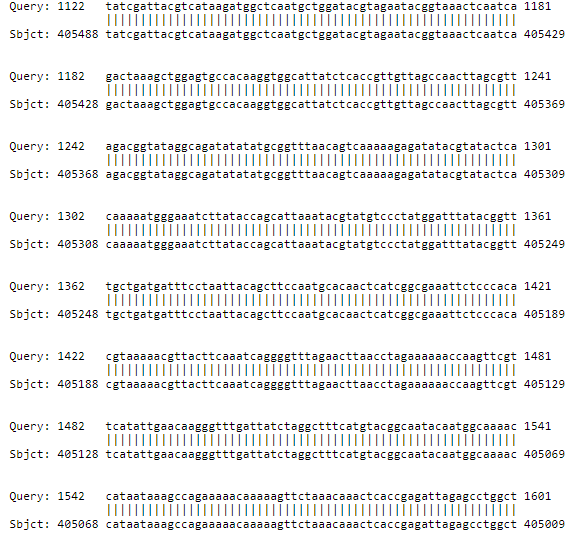


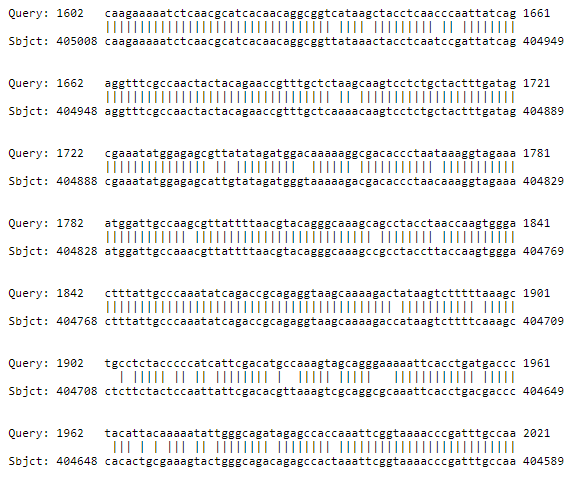


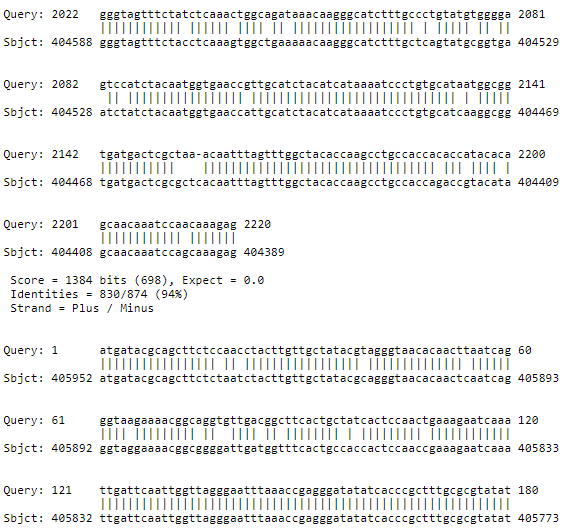


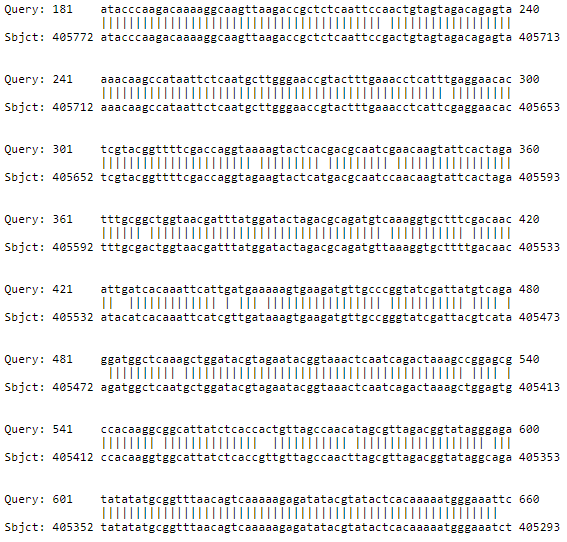


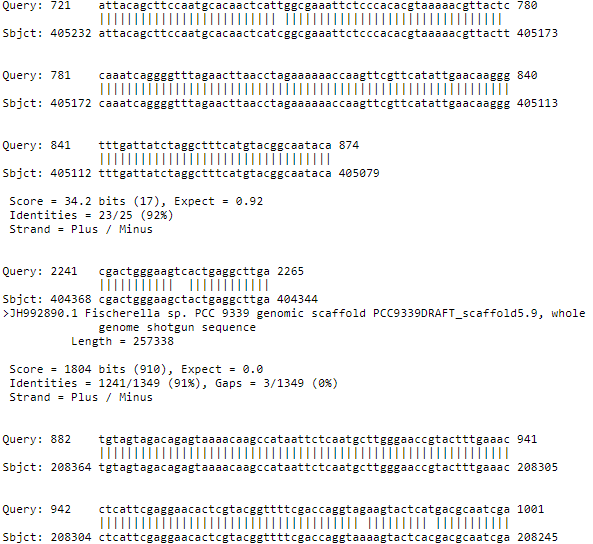


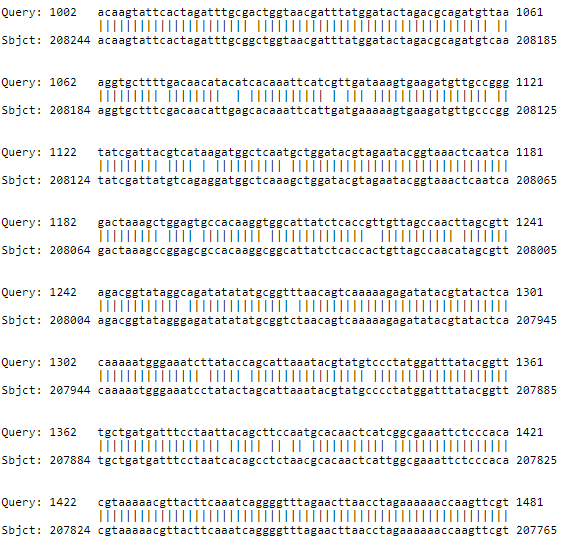


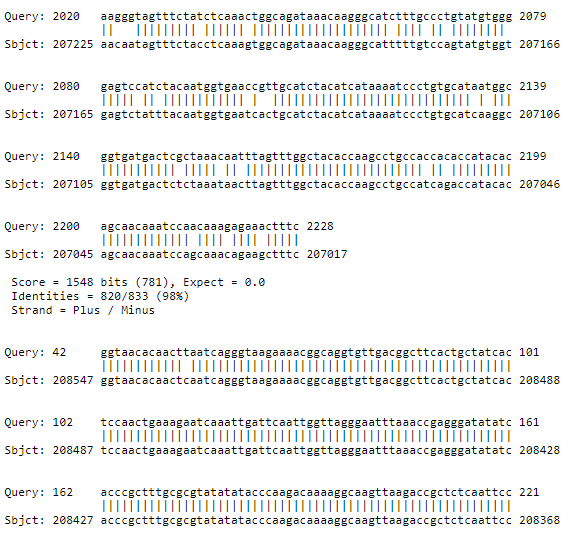

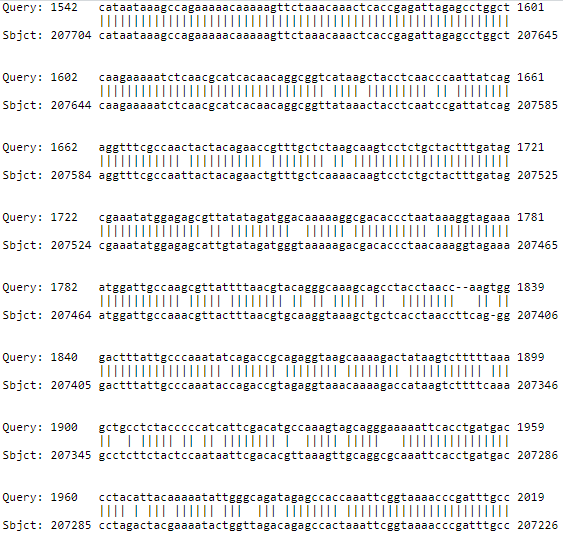


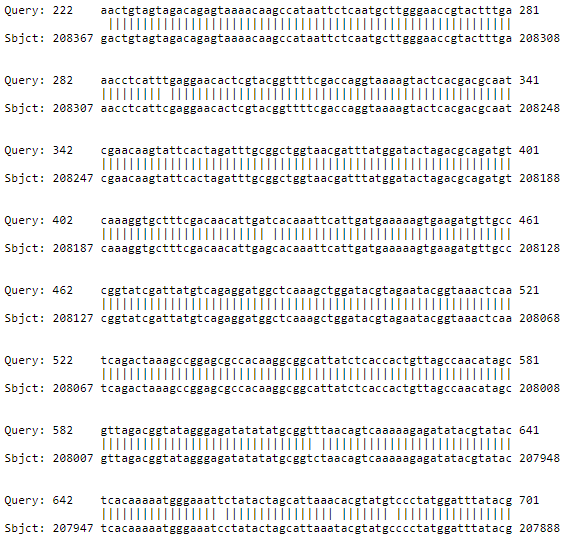

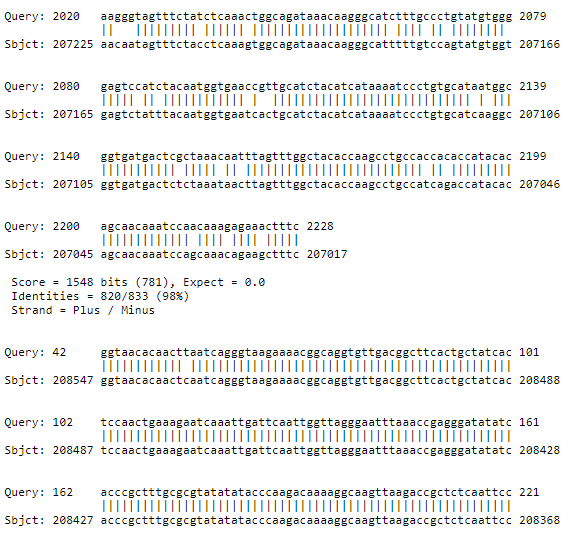


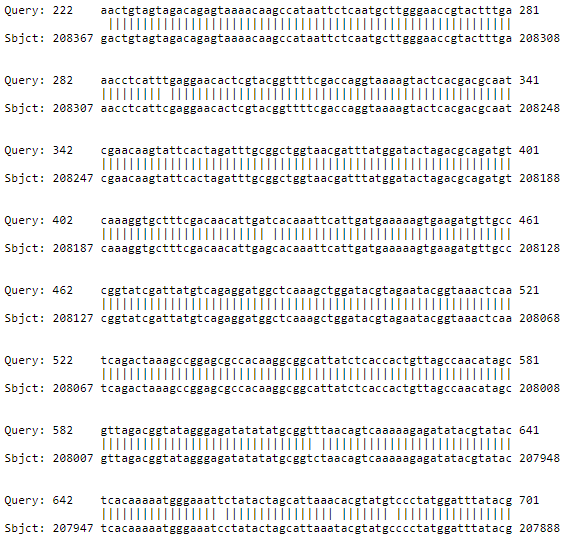


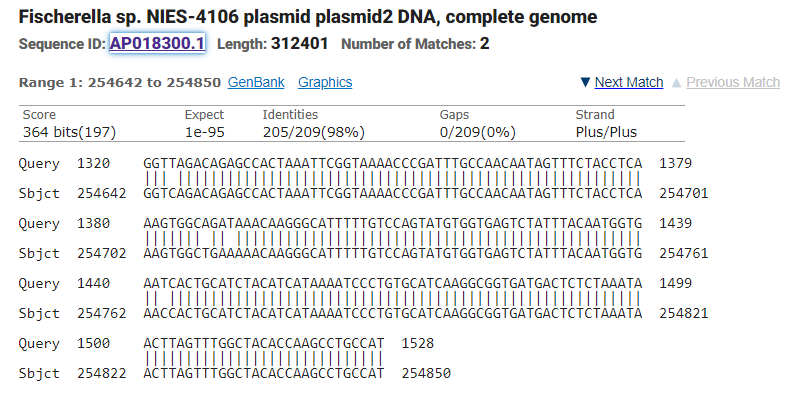

Supplement: Supplementary Table 6 — Blastn search between the “functional group II intron reverse transcriptase/maturase” gene (BLD44_007940) and the PCC 9339 genome and its plasmids. The blast result points to the 404344-to-406081 region of plasmid JH992892.1 containing a frame-shift leading to a pseudo-gene. The gene ID is RS35460 (PCC 9339). This gene is absent in PCC 7120, NIES-3754, and other cyanobacteria. Only a rudimentary copy of this gene is present in NIES-4106 plasmid DNA. [file Table_6.DOCX]
